# Hamstrings force-length relationships and their implications for angle-specific joint torques: a narrative review

**DOI:** 10.1186/s13102-022-00555-6

**Published:** 2022-09-05

**Authors:** Eleftherios Kellis, Anthony J. Blazevich

**Affiliations:** 1 Laboratory of Neuromechanics, Department of Physical Education and Sport Sciences at Serres, Aristotle University of Thessaloniki, TEFAA Serres, 62100 Serres, Greece; 2 Centre for Human Performance, School of Medical and Health Sciences, Edith Cowan University, Joondalup, 6027 Australia

**Keywords:** Semitendinosus, Biceps femoris, Semimembranosus, Injury, Muscle mechanics, Biomechanics, Exercise

## Abstract

Temporal biomechanical and physiological responses to physical activity vary between individual hamstrings components as well as between exercises, suggesting that hamstring muscles operate differently, and over different lengths, between tasks. Nevertheless, the force-length properties of these muscles have not been thoroughly investigated. The present review examines the factors influencing the hamstrings’ force-length properties and relates them to in vivo function. A search in four databases was performed for studies that examined relations between muscle length and force, torque, activation, or moment arm of hamstring muscles. Evidence was collated in relation to force-length relationships at a sarcomere/fiber level and then moment arm-length, activation-length, and torque-joint angle relations. Five forward simulation models were also used to predict force-length and torque-length relations of hamstring muscles. The results show that, due to architectural differences alone, semitendinosus (ST) produces less peak force and has a flatter active (contractile) fiber force-length relation than both biceps femoris long head (BFlh) and semimembranosus (SM), however BFlh and SM contribute greater forces through much of the hip and knee joint ranges of motion. The hamstrings’ maximum moment arms are greater at the hip than knee, so the muscles tend to act more as force producers at the hip but generate greater joint rotation and angular velocity at the knee for a given muscle shortening length and speed. However, SM moment arm is longer than SM and BFlh, partially alleviating its reduced force capacity but also reducing its otherwise substantial excursion potential. The current evidence, bound by the limitations of electromyography techniques, suggests that joint angle-dependent activation variations have minimal impact on force-length or torque-angle relations. During daily activities such as walking or sitting down, the hamstrings appear to operate on the ascending limbs of their force-length relations while knee flexion exercises performed with hip angles 45–90° promote more optimal force generation. Exercises requiring hip flexion at 45–120° and knee extension 45–0° (e.g. sprint running) may therefore evoke greater muscle forces and, speculatively, provide a more optimum adaptive stimulus. Finally, increases in resistance to stretch during hip flexion beyond 45° result mainly from SM and BFlh muscles.

**Supplementary Information:**

The online version contains supplementary material available at 10.1186/s13102-022-00555-6.

## Background

The hamstring muscles are a predominately bi-articular group consisting of the bi-articular semimembranosus (SM), semitendinosus (ST) and biceps femoris long head (BFlh) and the mono-articular biceps femoris short head (BFsh). The muscles therefore have important but variable effects on movements requiring hip and knee joint motion, and impaired hamstring functional capacity has been linked with ligamentous injuries [1], low back pain [2] and neuromuscular disorders [3]. Additionally, strain injury to the muscles themselves represent one of the most important and prevalent sport injuries, with both high injury and re-injury rates [4, 5].

BFlh is injured more frequently than the other hamstrings components [5–8], with different injury mechanisms potentially affecting each hamstring muscle [9]. Acute injuries during sprint running, for example, mostly involve BFlh and are attributed either to high peak knee flexion and hip extension forces [10, 11] or to the sudden activation of the hamstrings as they lengthen [12–15]. Simulation studies predict that the peak stretch magnitude is greater in BFlh than SM and ST during the swing phase of running [15, 16]. In contrast, stretch-type of injuries are thought to result from acute or repetitive overstretching of the hamstring muscles and predominately involve SM [17]. Further, recent reviews concluded that improvements in hamstring muscle strength, flexibility (i.e. voluntary, passive muscle elongation), and activation during activity varies between exercises [18, 19]. This indicates that hamstring muscles operate differently, and over different lengths, between movement tasks despite performing a similar role. To the best of our knowledge, the force-length relationships of individual hamstrings has not yet been determined.

Early studies suggested that hamstring injury was associated with a shift of peak torque at shorter angle lengths [20], which suggests that evaluation of the torque-angle relationship may be useful as a hamstring injury risk predictor and a return to play measure [21]. A more recent review, however, has raised doubts about the use of angle of peak torque [22], naming several limitations of knee flexion-–angle curve assessment, which relate to the potential influence of muscle architecture, neural activation, and moment arms as well as experimental limitations. Since the hamstrings are predominately bi-articular, it is unclear whether the range of joint angles used in various strength tests [23, 24] cover the full operating length of the hamstrings, and whether the shifts tend to occur only when the muscle is at a longer length and the passive elastic structures bear significant load, or whether alterations are also observed at the shorter muscle lengths common to many other activities. Further, it is not clear how a change in the joint torque-angle relationship reflects changes in the properties of each individual hamstring muscle, so it is not known whether the torque-angle effects are linked to the injured muscle specifically or are a symptom of another issue within the group.

To fully describe the hamstrings’ force-length properties, the joint torque exerted over a broad range of hip and knee joint angles should be recorded. In theory, the shortest length is achieved when the hip is fully extended and knee fully flexed. Thus, different combinations of hip and knee joint positions can result in the same hamstring length, so it is important to determine whether force production during contraction of each hamstring muscle is affected in relation to the muscles’ lengths or also by the respective joint angles. Hence, it is necessary to determine whether exercise training at a common hamstrings length but using different joint angles, as might be achieved using different exercises, leads to the same outcomes as far as hamstring functional adaptations and injury propensity. Further, when the hip angle is fixed, the hamstrings’ operating length depends on its initial length, as determined by the fixed hip joint angle, as well as the changes in length determined by the movable joint, i.e., the knee. In typical movements such as running or kicking, the hip and knee joint angles change simultaneously. Consequently, the muscle operating length range undergoes a continuous shift from one combination of hip and knee joint positions to another. Hence, conclusions regarding hamstring function that derived from a specific exercise movement pattern may not apply to other exercise conditions. This may lead to erroneous conclusions regarding the most optimum exercise stimulus for improving hamstring muscle function.

Description of the relationship between the active joint moment (torque) and joint angular position provides information about the effect of muscle length on force capacity; the generated torque around the joint reflects the interaction of the muscles’ force-length relationships, the joint moment arm, and the level of activation [25–27]. This interaction may differ between individual hamstring muscles owing to their differences in morphology, innervation, and architecture [28]. If each individual hamstring muscle works on a different region of its force-length relation at a given joint angle or range of motion, then different exercises may coopt different components of the hamstrings differently.

To begin to answer some of these questions, it is important to fully understand the force-length properties and moment arm profiles of the individual hamstrings components and how these are affected by relative changes in hip versus knee angles. While previous reviews have provided detailed examination of activation patterns during various exercises [18, 19, 29], the impact of architecture, activation, and moment arm and the resulting force-length properties of each hamstring component, and their subsequent impact on the knee flexor torque-angle relationship, have not been thoroughly investigated. The purpose of this review, therefore, is to examine the factors influencing the force-length and torque-angle properties of the hamstrings and to relate them to their in vivo function. Specifically, the following questions will be addressed: (1) What is the operating length that results in the greatest tension capacity of the hamstring muscles at the sarcomere/fiber level? (2) What is the optimal length for force generation of each component of the hamstrings, and the hamstrings as a whole? (3) How does joint moment arm interact with force and how does this impact experimentally-obtained knee flexion torque? (4) How does muscle activation capacity affect the torque-angle impact of each muscle and the hamstrings as a whole? (5) How does hamstrings-dependent joint torque, obtained during strength tests at various hip and knee joint angles, influence muscle force-length properties, moment arms, and activations within the hamstrings muscle group?

## Main text

### Literature search

The articles selected for review were obtained via searches of Sport Discus and MEDLINE between 1966 and January 2022. The keywords used in this search were ("hamstring" OR "knee flexor" OR "biceps femoris" OR "semimembranosus" OR "semitendinosus" OR “hip extensor”) AND ("torque" or "force" or “strength" OR "architecture" OR "force-length" OR "moment arm" or “lever-arm” or “mechanics” or “torque-angle” or “activation” or “neuromuscular”). From the 6741 abstracts returned, full-text articles were included for review if they were related to hamstring force generation properties. The reference lists of included papers were also interrogated to detect other relevant papers that might not have been found in the search. Articles were included in the analysis if they reported measurements of hamstrings force or torque or moment arm or activation in relation to joint angle or muscle length during isolated knee or hip joint movements in the sagittal plane.

### Length-tension and force-length relationships in hamstring muscles

#### Active tension: experimental observations

The length-tension properties of skeletal muscle fibers have been described at the sarcomere level [25, 30–32]. Nevertheless, the validity of applying these data to the estimation of force-length properties of whole human muscles in vivo is questionable [26, 31]. Reasons for incongruities between sarcomere length-tension and whole muscle force-length relationships include: (1) sarcomeres within a fiber may have different rest lengths and work at different lengths during contraction [33]; (2) sarcomeres near optimum length at a given muscle force will contribute more to fiber force than other sarcomeres in the series that are at suboptimum lengths, so the force in a fiber may be higher than expected by estimation from the mean sarcomere length and closer to the force of those sarcomeres at optimum length [26]; (3) fibers attach at angles to the tendon and therefore contribute less force than expected (to the cosine of fiber angle), and this angulation changes with muscle length and varies both within and between muscles [28, 34]; (4) fibers rotate during shortening (and rotate in fixed-end isometric contractions due to stretch of series elastic components) so fiber length does not change in a 1:1 relationship with muscle length as the joint is moved or as force increases during contraction [35, 36]; (5) different regions within muscles, which contain fibers at different relative sarcomere lengths, may be activated more or less than other regions within muscles, so muscle force may be more affected by a muscle compartment in which the fibers are working at specific (optimum or sub-optimum) sarcomere lengths [33, 37]; and (6) different muscles within a synergist group, which may possess sarcomeres operating at different lengths to the others, can be differentially activated, so the output of the group is not equal to the combined, estimated output of each muscle within the group [26, 38]. Therefore, it cannot be assumed that the sarcomere length-tension relationship is directly aligned with the force-length relationship of a muscle, or that the sum of expected forces produced by each muscle is equal to the whole muscle group output, during voluntary contraction.

Active tension is usually defined as the force generated by active processes which require energy [31]. To the best of our knowledge, the length-tension properties of sarcomeres or fibers within human hamstrings muscles have not been previously reported. For the hamstrings, the in vivo length range during joint rotation (i.e. the muscle operating range) [39] is defined by the simultaneous changes in hip and knee angles. Chleboun et al. [40] estimated the BFlh sarcomere length-tension relation based on fascicle length measurements (using ultrasound imaging) at various joint positions and using cadaveric reference data. Estimates were made possible after applying corrections for sarcomere shrinkage due to embalming and for the elongation of the tendon and aponeurosis. It was estimated that BFlh worked on the ascending limb of the sarcomere length-tension relation when the hip was in the neutral position (hip flexion angle = 0°) and the knee extended from 90° to 0° of knee flexion, but on the descending limb when the hip was flexed to 90° [40]. Further, it may be relevant that hip angle changes were found to influence BFlh sarcomere length more profoundly than knee joint angle changes. In a more similar study, Cutts [41] measured the sarcomere lengths of several muscles in three cadavers using laser diffraction and predicted the operating length range based on muscle-tendon unit length changes and pennation angle of each muscle. Based on these estimates, SM and ST were found to work on the ascending limb of the sarcomere length-tension relation when the hip was in the neutral position and the knee flexed 130°, but on the descending limb when the hip was flexed to 90° and the knee only slightly flexed (13°). Further, BFsh operated mainly on the ascending and plateau region of the length-tension relation. However, these data were captured at rest, which may not allow description of sarcomere lengths during active contraction when series elastic components are stretched, muscle fascicles rotate away from the line of action of the muscle, and potential regional activation differences reduce energetic isotropy within the muscle. In addition, in the study by Chleboun et al. [40] hip and knee joint ranges of motion were restricted to 90°, and thus the effects of angles greater than 90° on the length-tension relation were not examined. Similarly, Cutts [41] provided predictions of length-tension relations based on estimates at two joint positions and assumed that tendon strain was negligible. Regardless, the current evidence suggests that hamstrings muscles often work on their ascending limb and plateau region of their force-length relations, only working on the descending limb when the hip is flexed and the knee relatively extended.

**Fig. 1:**
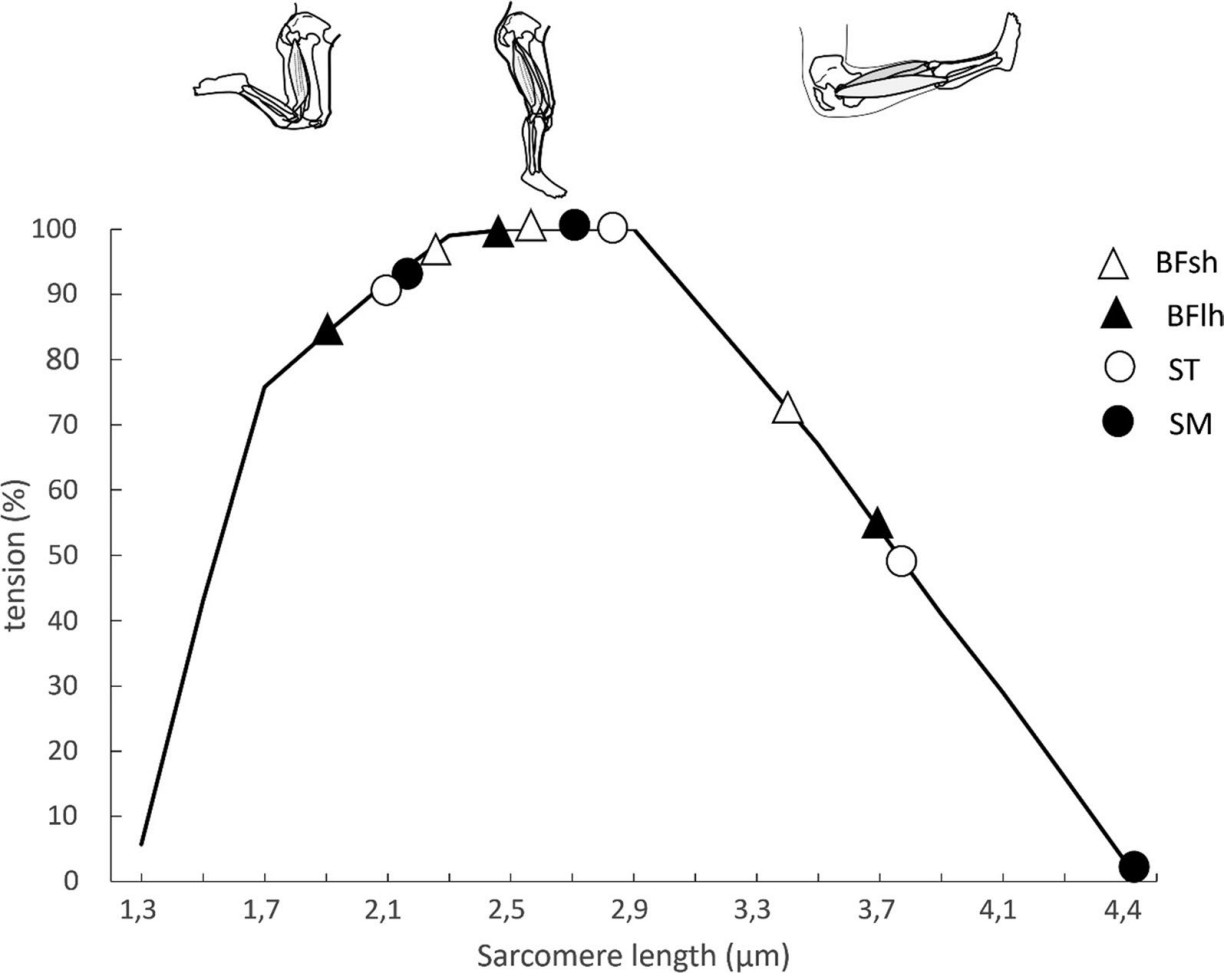
Illustration of length-tension data in the hamstrings, as reported in the literature. Sarcomere lengths for each of the four hamstrings muscles at three different joint angle configurations (shown in images above graph) were estimated based on cadaveric measures, in vivo measurements of muscle-tendon [41], or fascicle lengths [40] and after taking into consideration architecture and tendon properties

**Fig. 2:**
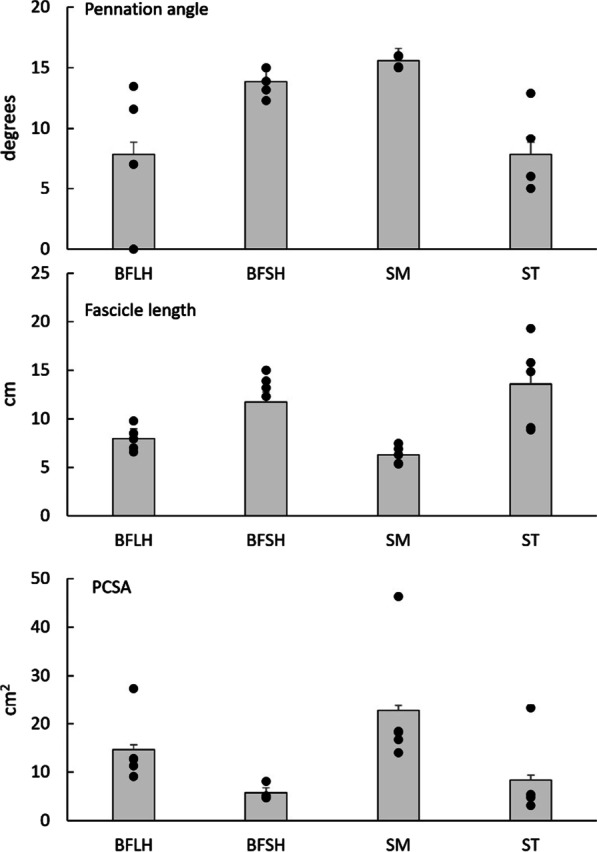
Mean pennation angle, fascicle length and physiological cross-sectional area (PCSA) of biceps femoris long (BFlh) and short head (BFsh), semimembranosus (SM) and semitendinosus (ST). Data were obtained from Kellis et al. [42], Wickiewicz et al. [44], Friedrich and Brand ^[45]^ and Ward et al., [43] from a total of 34 specimens (13 females, 12 males and 3 of unspecified Sex, age 62.7 years, body mass 77.56 kg and height 171.67). Error bars indicate standard deviation and black color circles represent mean values reported by each study

Figure 1 summarizes the operating length ranges provided by previous studies [40, 41] and shows that SM works over a broader range of sarcomere lengths compared to ST, BFsh and BFlh (Fig. 1). These differences may be related to the architectural properties of each muscle. In particular, the length-tension relation of an isolated muscle is qualitatively determined by its architecture [25]. For example, muscles with greater PCSA have a greater force-generation capacity while muscles with longer fibers have a greater excursion capacity [25]. In the hamstrings, important architectural differences exist between individual muscles [9, 28]. Hamstring architecture shows large variations in the literature, which results from morphological variations within and between each muscle as well as methodological differences between studies (for a detailed review see Kellis [28]). Figure 2 presents average values for basic architecture parameters from four cadaveric studies [42–45]. These studies show that ST has a smaller PCSA and, therefore, a lower maximum force generation capacity than BFsh, BFlh, and SM. SM has the greatest PCSA of all hamstrings so it should be considered a potentially important contributor to overall muscle force. Both BFlh and SM are more pennate than ST [28, 42, 43] and should thus generate more force for a given muscle volume since fiber angulation increases PCSA for a given volume, while subsequent fiber rotation during contraction might allow fibers to work at slower contraction velocities than would otherwise be required, shifting the power-velocity relation towards faster speeds [46]. On the other hand, ST has almost twice the fiber resting length-to-sarcomere length ratio as BFlh and SM [42], so its sarcomeres likely operate at a shorter length, and if all muscles experience the same absolute change in fiber length then the long ST fibers would display less sarcomere length change than those of BFlh and SM [47]. This is in line with length-tension predictions provided by cadaveric experiments [40, 41] (Fig. 1).

At the fiber level, it can be hypothesized that when ST operates on the descending part of its length-tension relation (i.e., at very long lengths), BFlh, SM and the monoarticular BFsh would be unable to produce meaningful contractile force and undergo greater relative strain due to extreme fiber elongation. Incorporating the information above, modeling based only on changes in fiber length and pennation angle during contraction indicate that most of the force at intermediate hamstrings lengths is produced by SM and BFlh due to their large PCSA, while ST typically produces smaller forces but still retains some capacity at the shortest and longest muscle lengths [42].

#### Active tension: simulation studies

**Fig. 3:**
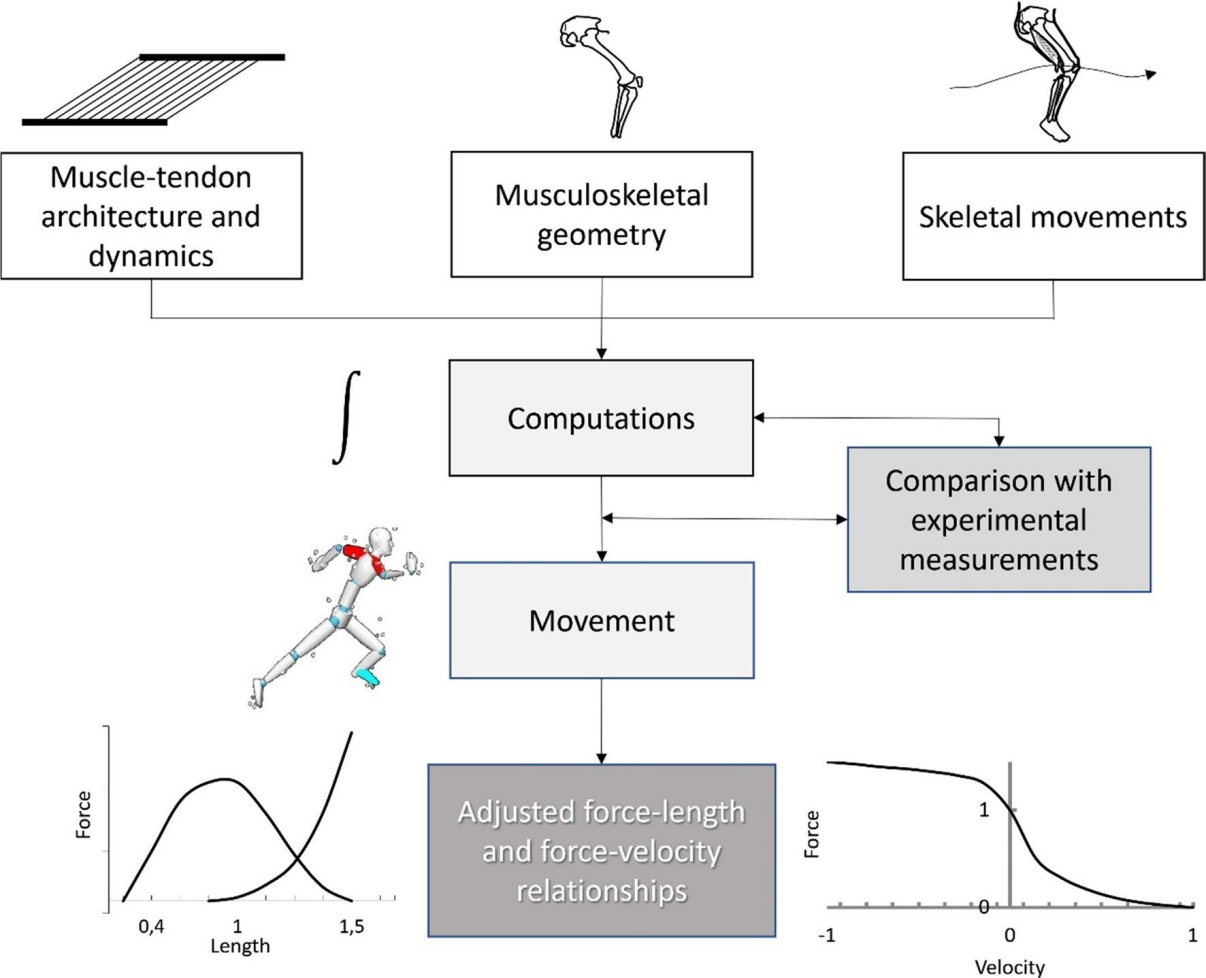
Schematic of a muscle-driven model. The model is used to predict the movement of a musculoskeletal system using data sets of experimentally measured muscle architecture and joint geometry as well as mathematical equations that define muscle-tendon (force-length, force–velocity, tendon properties) and skeletal movements [48, 50]. Muscle morphology data used in the models are mostly obtained from cadaveric data sets [43–45, 49, 55, 56], while in some cases they are combined with in vivo measurements (MRI) [51, 52, 57]. Subsequently, the model parameters are matched to experimental kinematic data which are collected during a particular movement (walking, for example) and are therefore adjusted so that they correspond to experimentally obtained ground reaction forces and moments [48]. Finally, algorithms are used to generate a set of muscle excitations that produce a coordinated muscle-driven simulation of the person’s movement [48]

**Fig. 4:**
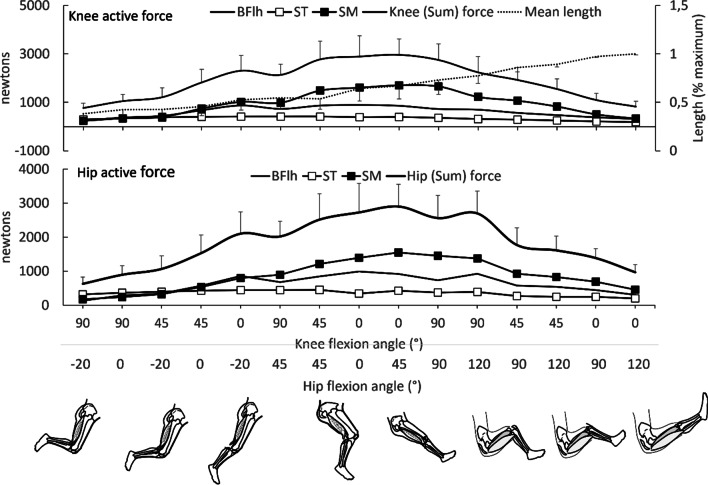
Mean (SD) active knee flexion (upper graph) and hip extension (lower graph) forces of biceps femoris long head (BFlh), semitendinosus (ST) and semimembranosus (SM) at 15 different hip (H) and knee (K) flexion angles as predicted using forward simulation modeling. Individual muscle forces and mean fiber lengths are also presented. Joint positions are arranged from left to right, from shorter to longer muscle lengths. Values were obtained from the Lower limb model 2010 [50], the full-body running model [53], the refined musculoskeletal model [51], the Gait2354_simbody model [49, 54] and the full body model [52] using OpenSim version 4.2 software [58]. Using each model, the hamstrings muscles were fixed at five hip flexion angles (0° = neutral, −20°, 45°, 90° and 120°) and both active and passive forces and joint torques were computed at each 10° of knee joint motion from 0° (full extension) to 100° of flexion. Error bars indicate standard deviation

Although it is practically impossible to directly measure the active force-length properties of individual hamstrings components in humans, important information relating to their force generation properties can be drawn from computer simulations [48, 49]. Muscle-driven models are used to predict the movement of a musculoskeletal system by using data sets of experimentally measured muscle architecture and joint geometry and mathematical equations that define muscle-tendon (force-length, force–velocity, tendon properties) and skeletal movements [48, 49] (Fig. 3). These predictions are subsequently adjusted based on kinematic or/and kinetic data of a given movement. Because the muscle parameters are altered so that the model produces realistic outputs, the final muscle parameters are thought to reasonably reflect in vivo muscle parameters. To describe the predicted hamstrings force and torque generation properties using this process, we used five lower-extremity anatomical models [49–54] to simulate hip and knee joint motion of an average male and then presented the average predicted active fiber forces at different hip and knee joint force combinations in Fig. 4 [Additional file shows model characteristics in more detail (see Additional file 1)]. Consistent with the estimates based on muscle architecture (Sect. 2.2.1, above), ST showed a flatter active (contractile) fiber force-length relation than BFlh and SM, however the predicted optimal angle for contractile force production depended on the combination of hip and knee joint angles [Additional files show these data in more detail (see Additional files 2 and 3)]. Predicted ST force was relatively constant across the entire length range with force values appearing slightly higher at shorter lengths, either when hip angle was 0° with knee flexion angle 10–20° or when hip flexion was 45° and knee flexion 60–70° [50]. Nonetheless, its small PCSA ensures that it makes a relatively small force contribution throughout the muscle length range. For BFlh, the greatest force is exerted at longer lengths, from 45° hip flexion and a knee flexion angle of 10°–30° [50]. Τhe predicted optimal active capacity occurs at even longer lengths, from 90° hip flexion and 70°–80° knee flexion [50] to 45° hip flexion and 10°–30° knee flexion [50]. BFlh and SM provide the majority of force applied by the hamstrings through most of the length range of motion. For all muscles, these predictions are consistent with experimental observations that hip flexion angle changes cause greater fiber length changes (40–65%) than knee joint angle changes (25–45%). Thus, the outputs of optimized models are consistent with the conclusions of observational studies described in 3.1. above, and suggest that the hamstrings are excellent force producers at relatively long in vivo muscle lengths.

#### Passive tension

**Fig. 5:**
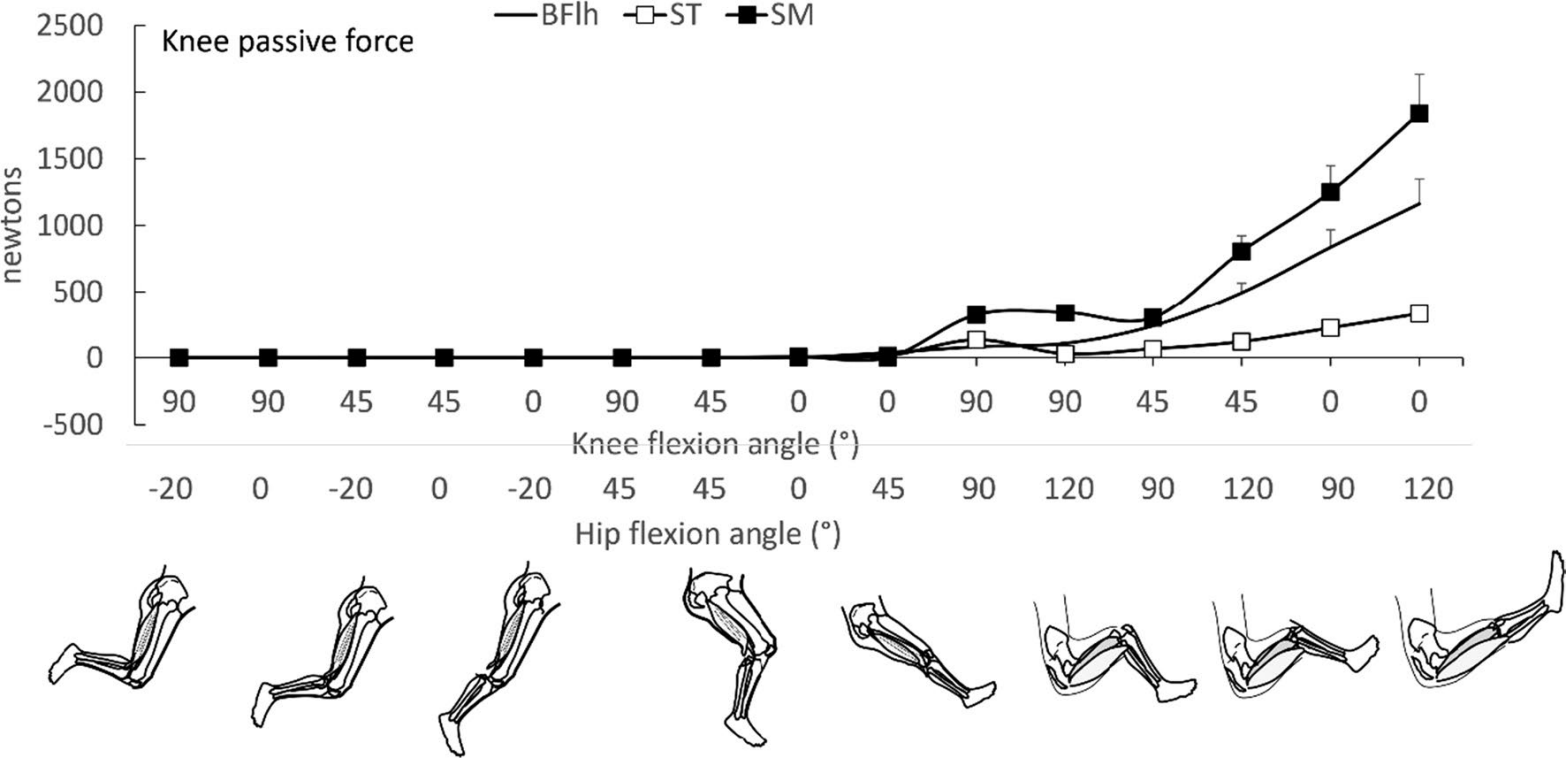
Mean (SD) passive forces of biceps femoris long head (BFlh), semimembranosus and semitendinosus (ST) at 15 hip (H) and knee (K) flexion angles predicted using forward simulation modeling (0° = neutral hip position, negative hip angle indicates extension; 0° = full knee extension). Values were obtained from five models [49–54] using OpenSim (version 4.2); for details see main text. Error bars indicate standard deviation

Passive tension refers to the force that is recorded when the muscle is stretched without activation [31]. This tension results from interactions between fibers, tendons, and aponeuroses, and is complex process that is still under investigation [59, 60]. It is therefore not a surprise that less information exists for the passive part of the length-tension relation in individual hamstring muscles. These data can again be estimated using the models described above. These models estimate that the predicted passive fiber force to resist elongation is almost three times greater in SM and BFlh than ST, as shown in Fig. 5. Nevertheless, the point at which the passive fiber force begins to contribute tension, often defined as slack length, occurs at similar hip flexion (45°) and knee (30–40°) angles for all muscles. In vivo estimates (using ultrasound) appear to enforce these predictions, as BFlh fascicle length increases substantially during passive motion where the hip flexes from 45° to 90° or more and simultaneously the knee extends from a flexion angle to full extension [36]. Modelling results, however, should be treated with some caution. A recent study [61] compared the shear-elastic modulus of ST (measured using shear-wave ultrasonography) with passive fiber force that was estimated using two mathematical models [49, 52, 54]. Their results showed that the joint angle of passive force onset differed compared to the angle of shear-wave elastic modulus onset. This discrepancy may be attributed to limitations in mathematical modelling approach where passive force onset is frequently set at the optimal fiber length [49, 52, 54, 61] even though this is not always a valid assumption [60]. Further, in most cases, passive forces are calculated as the difference between total and active forces, which is also incorrect [31].

Tendon compliance can influence a muscle’s length-tension properties. A stiffer tendon, for example, will exhibit less length change as muscle force changes during contraction over a full range of motion, thus reducing muscle length variation. However, it will also reduce the overall length change of the muscle and/or velocity of shortening during stretch-shortening cycles since the muscle does not need to compensate for large tendon stretch [62]. To the best of our knowledge, no studies have directly measured tendon/aponeurosis stiffness in hamstring muscles. I*n vivo* estimation of distal tendon-aponeurosis complex strain (using ultrasonography) has shown that distal tendon-aponeurosis strain of all hamstrings does not change significantly during a 45° range of motion knee extension [63]. During a 90° passive knee extension, however, BFlh distal tendon-aponeurosis strain is much greater than ST [64]. These results, however, have limited value for understanding the influence of tendons and aponeuroses on hamstring muscle length-tension properties, because neither the proximal tendon or aponeurosis properties were measured nor the tissue stiffness quantified. In addition, there is evidence that stiffness varies significantly along the tendon and aponeurosis [65]. This indicates that stiffness measurements taken from one particular tendon-aponeurosis segment may not be representative of the properties, and thus the effect, of the tendon-aponeurosis unit on the length-tension properties of the muscle.

The tendon length-to-muscle fascicle length ratio is also considered an important index of architecture and which is related to muscle-tendon unit function [66, 67]. Assuming a constant elastic modulus and PCSA, the larger the tendon length-to-muscle fascicle length ratio, the longer the tendon relative to its fibers, and the more compliant the muscle-tendon unit [28]. Muscles with relatively long tendons are more suitable to store and release a maximum amount of elastic energy whilst muscles with relatively short tendons tend to generate high force and maximal shortening velocity and thus produce substantial work and power [67]. To the best of our knowledge, no studies have examined differences in this ratio between the hamstring muscles. Nevertheless, Delp and Zazac [68] used data from cadaveric studies [44, 45] and reported a tendon length-to-muscle fascicle length ratio of 4.5 for SM, 3.1 for BFlh, 1.3 for ST and 0.6 for BFsh. We also calculated this ratio using the cadaveric dataset published more recently by Woodley and Mercer [69] and the values were 11.6 for SM, 7.44 for BFlh, 3.7 for ST and 0.9 for BFsh. Even though the size of the ratio differs between quite notably these studies, it appears that SM shows almost 1.5 and 3 times greater tendon: fiber length ratio than BFlh and ST, respectively [66, 68]. Compared to other muscles such as the tibialis anterior or gastrocnemius, the hamstrings could be characterized more like force/work producers and power generators [66, 68]. Within the hamstrings, SM seems to be designed for tasks in which large amounts of energy need to be temporarily stored in its relatively long series elastic component (tendons and aponeuroses) [67]. In contrast, ST and BFsh may generate high force and maximal shortening velocity and they are able to produce maximal muscle work and power [67].

While the relation between changes in muscle-tendon unit length and individual muscle forces during passive joint movement has not been documented, several studies have reported hamstring stiffness during passive joint motion. Magnusson et al. [70] used a geometric model alongside experimental measurements to predict that BFlh itself would have the greatest stiffness and ST the lowest during a slow stretching maneuver. Using shear-wave ultrasonography, recent studies have reported that SM shows the greatest shear-wave modulus (and hence, greater stiffness when considering its large CSA) and ST the lowest during passive stretching [65, 71–77]. BFlh elastic modulus appears to be lower than SM but greater than ST [65, 71–77]. Shear-wave elastography measurements, however, have some inherent limitations, including that the shear elastic modulus does not provide information about the amount of muscle elongation [78] and that it is highly dependent on measurement location [65]. Finally, Kumazaki et al. [79] measured muscle fascicle length and passive changes in muscle-tendon unit length in cadavers and reported that the change in fascicle lengths in SM and BFlh were greater than changes in ST and BFsh. Based on this information it can be concluded that, due to its high CSA and size, SM displays the greatest resistance to stretch amongst the hamstrings.

Based on the above, several issues arise regarding the hamstrings’ force-length relations that remain unclear. First, the in vivo force-length relationships have not been experimentally obtained using the whole range of hip and knee flexion angles, and, hence, force production through the full hamstrings operating range remains unclear. More research is also necessary to determine the effect of pelvic and tibial rotation on the hamstrings’ force-length relationships [80]. Second, most of these conclusions are drawn from limited experimental evidence. Third, information from forward simulation models is useful but outputs are susceptible to the effects of assumptions while information regarding the passive elements of the muscle-tendon unit need to be treated with caution. Finally, limited experimental evidence exists describing SM and BFsh mechanical properties and that of their tendons; hence, the role of these muscles is mainly based on estimates provided by muscle-driven simulation models. Thus, more research is necessary to understand individual hamstring muscle passive force-length properties.

As stated at the beginning of this section, the relation between sarcomere length-tension and whole muscle force-length relationships is not as simple. Fascicle rotation during contraction results in a reduced shortening velocity of the fascicles relative to the belly shortening (often described as muscle gearing) [81, 82]. The influence of muscle gearing should be small in relatively parallel fibered muscles, such as ST and greater in more pennate muscles such as SM, BFlh and BFsh. Since muscle fascicle forces decrease with increases in shortening velocity, it can be expected that muscle gearing would result in a reduced force-generating requirement of the fascicles for a given muscle force in the pennate hamstrings components. Nevertheless, the magnitude of this effect depends on how the muscles change shape relative to the muscle’s line of action [82]. Azizi and Brainerd [82] concluded that changes in muscle shape in pennate muscles vary in a way that a pennate muscle can shift from a high gear during rapid contractions to low gear during forceful contractions. Thus, the force exerted by the pennate hamstrings’ components should vary depending on contraction load, and this may impact the reported force-length properties of the hamstrings as described in the present review. The magnitude of this effect, however, has yet to be described and is worth investigating in the future; although fascicle behaviors have recently been examined in BFlh [36, 83,84], muscle or region-specific length changes have not been monitored in order to determine the muscle’s gear during contraction.

Changes in 3D muscle shape during various activities can also influence the force generated by one muscle as well as its surrounding muscles, as surrounding muscles transfer transverse forces to muscles, thereby compressing them [85]. For the hamstrings components, which surround each other along their path and have tendinous inter-connections, force capacity may be reduced due to compression applied from one muscle onto the others. The precise impact of this effect is difficult to describe, as this requires detailed consideration of the 3D shape and architecture of each muscle and their possible connections (common tendon, for example) as well as changes in their 3D shape under various experimental conditions [86]. Nevertheless, there is evidence that quadriceps’ muscle force was lower when the muscles contracted as a bundle than the summation of their individual muscle forces if they had contracted in isolation from each other [87, 88]. Thus, simply adding individual hamstring forces, as is shown in Fig. 4, may result in an overestimation of actual muscle group forces. In addition, the influence of transverse forces and inter-muscular pressures will impact both the magnitude of force as well as the shape of force-length relation of each individual component within the muscle group [86].

#### Implications

Predictions based on anticipated changes in length have shown that ST may exert proportionally less force in a lengthening contraction than BFlh or SM, primarily as a consequence of it lengthening over a smaller distance as joint angles change [89]. Nonetheless, the above interpretations firstly assume that the contraction is purely eccentric and that there is considerable cross-bridge cycling. This approach, however, does not take into consideration the muscle force enhancement that occurs when the muscle is stretched whilst activated [90]. Shim and Garner [91] reported a 4.6% residual force enhancement (after stretch) during isometric flexion contractions at long muscle lengths (70° knee flexion) but not at short lengths (10° knee flexion) whilst Chapman et al. [92] reported a greater force enhancement at 30°and 60° knee flexion (8.9%) which increased further during submaximal contractions (39%). Hence, when the hamstrings work eccentrically during sprinting or kicking, they should show a significant force enhancement response (although only two studies have examined it; see [93]), which is presumably consistent across muscle lengths if working near or longer than the optimum length [94]. This force cannot be explained by traditional force-length data and, hence, hamstring muscle force descriptions based on this relation must be treated with caution. Secondly, the predicted passive forces do not contribute directly to eccentric force, i.e. they do not perfectly add to the active force, since the parallel elastic components shorten as the contractile element shortens with increasing muscle force (stretching the series elastic components, including the distal tendon) and therefore the passive force contribution at a given muscle-tendon length will be far less than predicted when the muscle is active rather than passive [95, 96].

In knee flexion exercises performed with a fixed hip angle (“knee dominant” exercises) the operating range depends on that hip angle. During leg curls or Nordic hamstrings exercises (where hip angle is ~ 0–15°), for example, the hamstrings would operate at shorter than optimal lengths as the knee rotates from 90° to 45° and reach their maxima (and close to optimum) as the knee rotates from 45° to 0° flexion. Recent measurements (using ultrasound) have confirmed that BFlh fascicles work at longer lengths at the terminal phase of the Nordic exercise [97]. When knee flexion is performed with hip angle 90°, the operating length starts longer than optimum at knee angles 0°–45° and reaches optimum as it flexes 45°–90°. Finally, during knee flexions performed with hip flexion angles > 90° (whilst in a seated position, for example) the muscles will work on the descending limb of their force-length relation unless the knee is flexed to < 90° (Fig. 4). In exercises in which hip angles change while the knee angle is fixed in relative extension (~ 0–15° knee flexion; “hip dominant” exercises), the limited evidence available indicates that the hamstrings can operate at optimal lengths for hip force generation at angles of ~ 45–90° of flexion. This corresponds to the late lowering phase of the good morning exercise [98] where hamstring muscle lengths increase approximately by 11–12% relative to normal standing position [99].

During daily activities such as walking [100], jogging or the start or end of a sit-to-stand sequence in which the hip angle ranges 15–20° extension to 20–30° flexion and the knee angles range 50–60° to 10–0° (0° = full extension), all hamstrings components should operate on the ascending limb of their force-length relation (Fig. 4). The operating length of the hamstrings during sprint running is of particular interest because of its injury consequence [9]. In the swing phase of sprinting, the hamstrings first shorten through hip angles of 40° (flexion) to 25° (extension) and knee flexion angles ranging 40–110° of flexion and then resist stretch through hip flexion angles 50–70° flexion and the knee extending to angles ranging 40–20° [16]. Studies using experimental measurements and simulation models have estimated that the hamstrings muscle-tendon units shorten and then lengthen approximately by 10–12% during sprinting [16, 101, 102] (relative to upright standing position), while fiber strain is 2–3 times greater [101] (for a review see Huygaerts et al. [9]). Therefore, during the early swing phase the hamstring fibers operate on the ascending limb of their force-length relations, and they then produce force at near-optimum lengths during the late swing (Fig. 4); even if the muscles lengthen further due to rapid knee extension (relative to hip extension) during the late swing phase, it is unlikely that they operate far down their descending limbs. Hence, muscle length alone is unlikely to be a factor affecting injury under most conditions. Since ST has a flatter relation, its force loss is less than in BFlh and SM (Fig. 4).

With the knee extended, passive resistance to stretch during hip flexion (lengthening) starts to increase from about 45° of hip flexion and is provided by all hamstrings. As the hip flexes beyond 45°, passive resistance increases almost three-fold (relative to neutral position) and mainly results from SM and BFlh resistance to elongation. Owing to its longer fibers, ST can operate over a greater range of motion without over-stretch. Hence, upon contraction, BFlh and SM fibers are predicted to work at longer lengths than those of ST (although, see issues regarding passive force estimation above). Interestingly, owing to its greater tendon length-to fiber length ratio and PCSA, SM is a very strong muscle and one that should store-release elastic energy, thus contributing a lot at the end of the recovering phase of sprinting as well as storing elastic energy.

### Moment arm

**Fig. 6:**
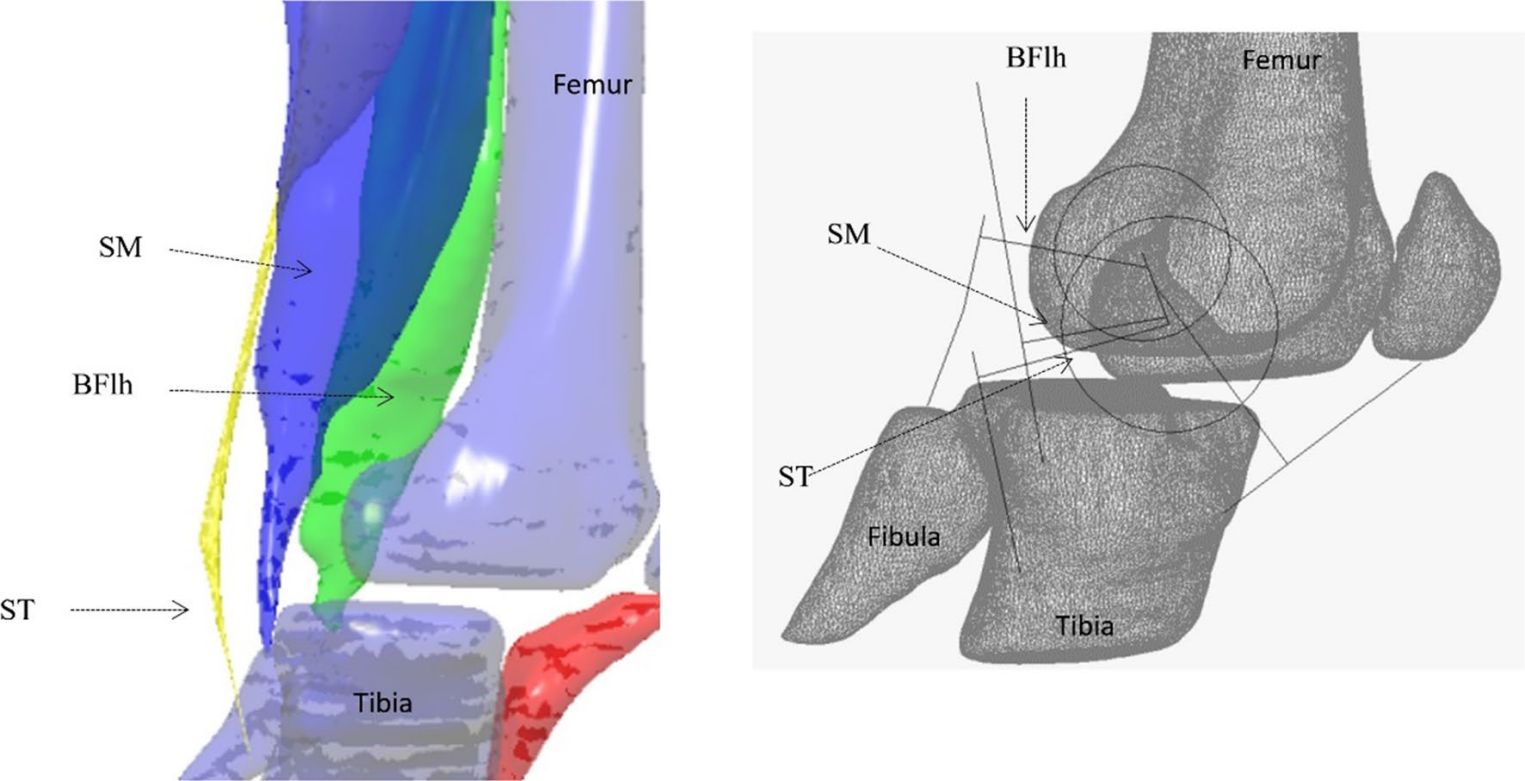
Morphology (left image) and moment arm (right image) of biceps femoris long head (BFlh), semitendinosus (ST) and semimembranosus (SM) about the knee joint. The images were collected using magnetic resonance imaging with the knee in slight flexion and the participant at rest. Images were then reconstructed using finite element analysis [104]

A muscle’s force contribution to joint torque is proportional to its moment arm length (Fig. 6). For the same muscle force, a muscle with a longer moment arm contributes more joint torque than a muscle with shorter moment arm [103], however a given muscle shortening then also produces less joint angular excursion, and thus velocity, when the moment arm is longer. *Ipso facto*, a given joint angular displacement will induce a greater change in muscle length when moment arm is longer [103]. Given that three of the four hamstring muscles are bi-articular, the relative moment arms at the hip and knee joints will strongly influence muscle joint torque contribution as well as their effects on joint rotation and angular velocity.

During normal joint rotation, both the moment arms and muscle forces change through the range of motion and thus influence the "shape" of the torque-angle relation. It is therefore possible that the optimum joint angles for muscle force production and joint torque production do not coincide [105, 106]. Not only are three of the hamstrings muscles bi-articular, so their moment arms are influenced by changes in both hip and knee joint angles, but individual muscles have different attachment sites across the hip and knee and thus a different line of action of their moment arms. The moment arm is therefore an essential factor influencing the relationship between the muscle force-length relations and the overall torque-angle relation.

Indicative moment arm values for the hamstring muscles are presented in Table 1. Different methods have been used to quantify moment arms, including cadaveric measurements, tendon excursion measurements, estimation using imaging techniques (magnetic resonance imaging, video-fluoroscopy, X-rays), and predictions from mathematical modelling. Despite between-study variations in moment arm values, hamstring muscle moment arms are generally found to be greater at the hip than the knee. Thus, for the same level of muscle force, the hamstrings will generate a greater hip extension than knee flexion joint torque, i.e., they tend to act more as a force producer at the hip but contribute relatively more to range of motion and angular velocity at the knee. It also follows that a given hamstrings muscle length change will induce less sagittal angular displacement at the hip than the knee. Nonetheless, the smaller moment arm at the knee should also assist elastic energy storage in the hamstrings during lower leg deceleration (as the hamstrings resist hip flexion and knee extension) because the small moment arm provides the conditions for a high muscle force for a given knee joint torque, thus stretching the series elastic component, and particularly the long distal hamstrings tendons. Upon recoil, the tendon can contribute significantly to high-speed shortening, partly because tendon recoil capacity is much faster than the maximal possible muscle shortening speed and partly because the short moment arm increases joint angular velocity for a given muscle-tendon unit shortening speed. Of the three bi-articular hamstrings, ST has the longest moment arm at both the hip [107–110] and knee [50, 104, 110–113] (Table 1). Therefore, ST should generate a greater hip extension and knee flexion torques for a given muscle force, which may partly compensate for its relatively small PCSA. Also, for a given change in hip and knee angles it should undergo greater length change than BFlh and SM, consistent with its flatter force-length relation and longer fiber lengths. Nonetheless, SM and BF (BFlh and BFsh) may experience better conditions for elastic energy storage-reuse, if their series elastic components are sufficiently long and extensible to allow it (and this appears to be the case for both muscles; reviewed in Huygaerts et al. [34]), owing to their shorter moment arms. In addition, it has been suggested that BFlh exhibits a larger moment arm at the hip than at the knee (Table 1) and therefore possesses a greater mechanical advantage at this joint. As a result, BFlh undergoes significantly more shortening during hip extension than knee flexion [19].

**Table 1:** Moment arm (MA) values (mm) of the hamstrings and angular position at which the moment arm was measured as they appear in the literature. Values in parentheses indicate the joint flexion angle (hip neutral position = 0°, knee. full extension = 0°)

Study	N	Age (years)	Mass (kg), Height (cm)	Method	Joint	ST		SM		BFLH	
						MA (mm)	Angle (°)	MA (mm)	Angle (°)	MA (mm)	Angle (°)
Arnold et al. [113]	3	-		MRI Cadavers	Hip	66.7	30	55.5	30	62.2	30
Thelen et al. [109]	14 (9F, 5 M)	18.2–19.6	65.7–84.7, 176.4–182.2	Cadavers	Hip	65*	40–50	50*	30	58*	30–40
Dostal et al. [114]	1			Cadavers	Hip	57	0#	46	0#	58	0#
Duda et al. [115]	6 (4F, 2 M)	67–79		Cadavers	Hip	44	0#	38	0#	43	0#
Visser et al. [116]	6 (5F, 1 M)			Cadavers	Hip					80*	80
Schache et al. [117]	4 M	26.3 ± 7.5	62.8 ± 8.7, 173 ± 4.2	MRI model	Hip					65–68	45–50
Trinler et al. [110]	10 (5F, 5 M)	28 ± 5	69 ± 12, 172 ± 0.8	Model (OpenSim)	Hip	75	30–40	60	30–40	70	30–40
Nemeth and Olsen [118]	10 (5F, 5 M) 20 (10F, 10 M)	78–82 63–70	160–176 589–736 N, 166–176	CT, Cadavers	Hip, all muscles		80	40			
Kellis et al. [104]	10 (5F, 5 M)	42.20 ± 7.63	77.81 ± 8.89, 73.6 ± 0.68	MRI	Knee	39.2	30#	33.4	30#	30.9	30#
Wretenberg et al. [119]	20 (9F, 10MF)	27 ± 5	71 ± 12.0, 175.0 ± 0.09	MRI	Knee	38.8	60#	30.4	60#	24.4	60#
Herzog and Read [112]	5 (3F, 2 M)	79.2		Cadavers	Knee	65 *	130	25 *	30	27 *	80
Spoor and Van Leuven [120]	1Μ	89	156	MRI-tendon travel	Knee	52	65	40	10	22	60
Arnold et al. [113]	3			MRI	Knee	52	65	40	10	22	60
Arnold et al. [113]	3			Cadavers	Knee	55.3	60	46.4	50	37.2	50
Arnold et al. [50]	21 (12F, 9 M)	83 ± 9	82.7 ± 15.3, 168.4 ± 9.3	Model (data from [43])	Knee	48 *	50	35 *	40		
Buford et al. [111]	15			Tendon excursion	Knee	55.4	57	46.3	53	30	55
Thelen et al. [109]	14 (9F, 5 M)			Model ^	Knee	50	80	38	20	30	50
Navacchia et al. [121]	7	63 ± 8,	72 ± 9, 170 ± 9	Stereo-radiography	Knee	45*	50	55*	55	30*	35
Visser et al. [116]	6 (5F, 1 M)			Cadavers	Knee					10	
Nemeth and Olsen [118]	10 (5F, 5 M) 20 (10F, 10 M)	78- 82 63–70	160–176 589-736 N, 166–176	CT-Cadavers	Knee			10			
Duda et al. [115]	6 (4F, 2 M)	67–79		Cadavers	Knee	18	0#	22	0#	40	0#
Trinler et al. [110]	10	28 ± 5	69 ± 12, 172 ± 0.8	Model (OpenSim)	Knee	45	50	35	50	40	30
Schache et al. [117]	4	26.3 ± 7.5	62.8 ± 8.7, 173.0 ± 4.2	MRI-model	Knee					56–65	30–35
Snoeck et al. [122]	7 (4 M, 3 F)	70 ± 10		Cadavers	Knee	44	60–70				
Kellis and Baltzopoulos [123]	10 M	23 ± 1.5	74 ± 3.8, 174 ± 4	Fluoroscopy	Knee, All muscles			23.9	35		
Smidt [124]	26 M	28	82, 176	X-Ray	Knee, all muscles			41	45		
Herzog and Read [112]	5 (3F, 2 M)	79.2		Cadavers	Knee, all muscles			35	95		

*Based on visual inspection of published graphs; *ST* semitendinosus, *BFlh*  biceps femoris long head, *SM* semimembranosus

^Model based on data by Arnold et al. [113]

#Angular position where moment-arm was measured

Hip moment arm values for each muscle vary as a function of hip joint angle (Table 1), with the hip extensor moment arm generally reported to increase gradually from 0° to ~ 30–40° of hip flexion before decreasing at greater flexion angles [15, 113, 117, 118], with the exception of one cadaveric study reporting a linear increase with increasing hip flexion [116]. These results suggest a greater mechanical advantage of the hamstrings around the hip in mid-range hip flexion angles. Compared with the other hamstrings, ST has a longer moment arm through the range 0°-90° of hip flexion [109, 110, 113], therefore increasing torque contribution relative to PCSA, requiring greater shortening relative to joint angular rotation, and reducing joint excursion relative to muscle shortening. In contrast, having the shortest moment arm of the three bi-articular hamstrings, SM has the smallest maximum torque capacity relative to PCSA, requires less shortening per joint angle rotation, and produces more joint rotation relative to muscle shortening. These moment arm differences are consistent with the smaller PCSA but greater excursion potential of ST but larger PCSA and smaller excursion potential of SM, i.e., differences in moment arm tend to reduce functional differences that would exist due to their architectures alone.

Reported knee moment arm-joint angle relations for the hamstring muscles vary within the literature (Table 1). Most studies report that moment arm-joint angle relations for all components follow an ascending-descending pattern as the knee moves from full flexion to full extension [108, 112, 113, 120, 121, 123, 124]. Two studies used x-ray visualization of the knee in relatively young individuals and observed the hamstrings moment arm (considered as a single muscle) to occur near full extension (i.e. 25–40° knee flexion) [123, 124] while another study performed on cadavers of older individuals [112] reported a more flexed angle for peak moment arm (Table 1). To the best of our knowledge, peak SM moment arm occurs at 20–50° of knee flexion [108, 110, 112, 113, 120, 121] while peak ST moment arm occurs later in the range of motion, at 50–130° of knee flexion [108, 110, 112, 113, 120–122]. Based on these data, relative to their architectures, SM may impact joint torques more when in greater knee extension but ST when in greater flexion. As for BFlh, most studies have reported a peak moment arm at mid-range angles of 35–80° of knee flexion [50, 108–110, 112, 117, 120, 121], although some studies reported a relatively constant moment arm across joint angles [116, 119].

**Fig. 7:**
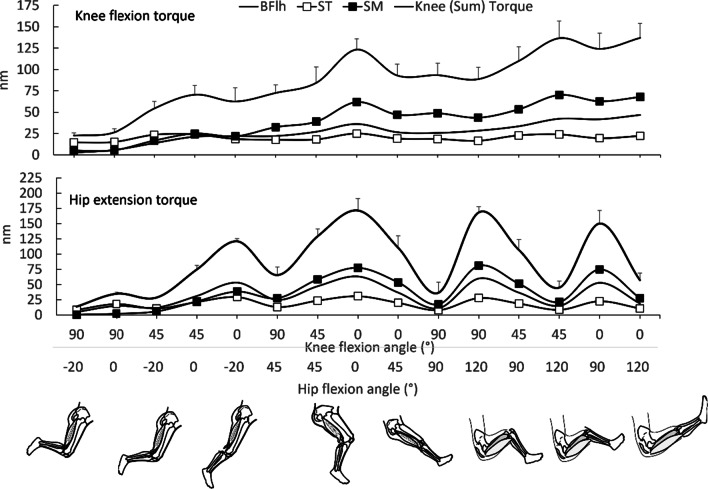
Mean (SD) knee flexion (upper graph) and hip extension (lower graph) torque contributions of biceps femoris long head (BFlh), semitendinosus (ST), and semimembranosus (SM) as predicted using forward simulation modeling. Individual muscle torques are also presented. Data are plotted for 15 hip (H) and knee (K) flexion angles which are arranged from left to right, from shorter to longer muscle lengths (0° = neutral hip position, negative hip angle indicates extension; 0° = full knee extension). Values were obtained from five models [49–54] using OpenSim (version 4.2); for details see main text. Error bars indicate standard deviation

The complex role of size, moment arm, and architecture of each synergetic hamstring component was recently confirmed [125] by estimating the torque generation capacity of each hamstring muscle during isometric efforts at 90° hip angle and 45° knee angle by combining in vivo PCSA (using ultrasound) and moment arm (using MRI) measurements. The product of PCSA and moment arm of ST was found to be smaller than BF (including BFlh and BFsh) and SM. However, the inter-relationships between force, moment arm, and torque for individual muscles are difficult to verify experimentally because of issues around measurement accuracy. In Fig. 7, the predicted torques of each individual muscle from our simulations are presented. Predicted torque increases at longer muscle lengths and is associated with an increase in moment arm of all muscles near knee extension. Further, comparison of the predicted active force (Fig. 4) with the torque-angle (Fig. 7) relations indicates that ST retains some torque capacity at intermediate lengths relative to BFlh (e.g. at hip angle = 45° and knee angle = 45°), which is related to the greater moment arm of ST relative to BFlh [Additional files show predicted moment-arm curves and peak moment for each model (see Additional files 4 and 5)]. Further, it is worth noting that at shorter lengths (e.g. at hip angles ≤ 90°) both ST and BFlh show similar torque capacity while SM is the main torque contributor (Fig. 7) while at longer lengths BFlh shows greater increase than ST, probably due to BFlh’s greater passive force (Fig. 5) [see Additional files 2 and 3].

Based on the above, and as shown in Table 1, moment arm values differ substantially between studies. This may be attributed to several factors. First, different methods have used to quantify the moment arm (see Table 1) and differences exist between moment arm values estimated in the sagittal plane [123, 124] and those estimated from three-dimensional reconstructions [104, 107, 109, 118, 119, 121]. Similarly, predicted values depend on the definition of the centre of joint rotation, including the instantaneous axis of rotation [50, 104, 109, 113, 124] or the tibiofemoral contact point [112, 119, 123]. Additionally, most reported moment arm values were obtained with the muscle at rest [50, 104, 109, 112–115, 119] or during submaximal contraction [117, 121, 123], however muscle contraction may alter the relative position of the tendon and the joint axis, thus influencing moment arm [126]. For example, Navacchia et al. [121] calculated a 30% difference in force estimation when using passive moment arm data (which is commonly used in models) versus data with muscles active. Further, the change in position between articular surfaces that occurs when the muscles are activated depends on the knee flexion angle, and this at least affects the shape of the BFlh force-length relation. These issues may lead to force magnitude estimation and force-length relation shape errors when using forward simulation or inverse dynamics methods [121]. Population characteristics also differ between studies, as cadaveric data were usually obtained from older individuals and these data cannot be generalized to younger populations. Hence, determination of the effect of moment arm on joint torque capacity or muscle force requirements is difficult and conclusions may vary markedly depending on which data set is used for modelling purposes.

#### Implications

SM has a longer moment arm than BFlh at the knee (it appears to be similar at the hip) and, hence, should have a greater knee flexion torque capacity but somewhat smaller joint excursion and velocity influence. Since SM also has a greater PCSA than BFlh and ST, it should be a very strong contributor to joint torque production (discussed below). Nonetheless, the greater force capacity and shorter moment arm of SM and BFlh should allow conditions under which elastic energy storage is significant, when compared to ST; they therefore may be able to participate successfully in high-speed and/or energy efficient tasks, including running. The long-fibered ST has the longest moment arm of the three bi-articular hamstrings muscles, indicating that torque-contribution limitations relating to its smaller PCSA may be partly overcome by its long moment arm, but also that its greater fiber length (sarcomere number) might be a requirement to overcome the larger excursion range imposed by the longer moment arm.

### Activation

Neural activation influences force/torque generation capacity and varies between conditions of muscle length, shortening speed and contraction type (eccentric, concentric, isometric). Hence, hamstring torque produced under different testing conditions is frequently attributed to neuromuscular activation factors [69, 127, 128]. Electromyogram (EMG) amplitude, measured using both surface and indwelling electrode techniques, has been primarily used to categorize muscle activation intensity, and therefore assist professionals when selecting the most appropriate exercise for recruiting a particular hamstring muscle (for relevant reviews see [19, 29]). However, the level of activity recorded using these techniques cannot immediately provide an estimate of the level of “activation”, defined as the activation state of the muscle between rest (0% active) and absolute maximum activation (100%); instead, it provides only a measure of the electrical activity recorded at an electrode under a prescribed set of conditions. Nonetheless, it is of interest to determine whether such data might provide meaningful information regarding muscle length-specific activation of hamstrings muscles. At a sarcomere or fiber level, the mechanisms influencing the relation between neural activation, force and length are complex and their examination is beyond the scope of this review [26, 31, 129]. In this section, therefore, we examine how hamstring muscle activity (EMG) varies as a function of hip and knee flexion angles as well as whether length-dependent differences exist in activation between individual hamstring muscles.

#### Hip extension contractions

A greater peak hip extension torque is also observed at more acute hip flexion angles [130]. During hip extension, the hamstrings act synergistically with other muscles such as gluteus maximus and the posterior head of the adductor magnus [131, 132]. Therefore, the relation between hip extension force and hamstring muscle activity is complex [133, 134]. Nevertheless, some studies have shown that hamstring EMG activity decreases as the hip is flexed towards 90° [135] but others have reported that the EMG signal is unaffected by hip flexion angle [130] (see Table 2).

**Table 2:** Methodological details and main findings of studies that examined the influence of hip and knee joint angle on muscle activities of the hamstrings during hip extension tests (hip neutral position = 0° in prone, unless stated otherwise, knee full extension = 0°)

	Participants		Normalization	Contraction			Angular position differences in EMG		
Study	(n, Sex)	EMG Type	(hip, knee angle)	(angular velocity, °/s)	Hip (°)	Knee (°)	BFlh	ST	SM
*Hip angle effects*									
Worrell et al. [130]	50 (25F, 25 M)	S	Max at any angle	ISOM	0, 30, 60, 90	90		NS	
Glaviano and Bazett-Jones [135]	22F	S	MVC	ISOM	0, 45, 90	0	NS	90 < 0, 45	
Coratella et al. [136]	10 M bodybuilders	S	MVC (0, 0)	Romanian, Step-Romanian, Stiff-leg deadlifts			Ascending > Descending phase	Ascending > Descending phase	
*Knee angle effects*									
Kwon and Lee [137]	20 M & F	S	MVC (-20)	ISOM	0	0, 30, 60, 90, 110	110 < 0	110 < 0	
Hahn [138]	18 M	S	MVC at each angle	ISOM leg press	Flexed	30–100, every 10°	60–100 < 30–50		
Kim and Park [139]	22 M	S	Raw	ISOM bridge		0, 60, 90, 120		60, 90, 120 < 0 120 < 60	
Lehecka et al. [140]	18 (16F, 12 M)	S	MVC (0, 45)	ISOM bridge		90, 135	135 < 90		
Sakamoto et al. [141]	31 (16 M, 15 M)	S	MMT (0, 90)	ISOM		0, 90		90 < 0	
Oh and Lim [142]	32 (14F, 18 M)	S	MMT (0, 60)	ISOM		H60K0 > H0K60	H60K0 > H0K60	H60K0 > H0K60	H0K60 vs H60K0

*BFlh* Biceps femoris long head, *ST* Semitendinosus, *SM* Semimembranosus, *M* Males, *F* Females, *HD* High Density, *S* Surface, *IM* Intramuscular, *HD* High density, *Norm* Normalization, *MVC* Maximum voluntary contraction, *H* hip angle, *K* knee angle, *NS* non-significant differences, *ISOM* isometric, *Isok* isokinetic, *ECC* Eccentric, *CON* Concentric, *NHE* Nordic exercise

Since the predicted force should increase when the hip is flexed from 0° to 90° (Fig. 4), then it does not appear that the EMG activation-length relation is synchronous with the predicted force-length relation. Further, hip extension contractions (mostly isometric contractions with the hip moving from 0 to 40° angle) with the knee in extension result in greater EMG activities than contractions with the knee flexed [137–141], which makes sense as the hamstrings operate at the plateau region of their force-length relation through these hip and knee ranges (Fig. 4) and produce greater force with the knee extended than flexed.

#### Knee flexion contractions—hip angle effects

Whilst an increasing hip flexion angle is associated with a higher peak knee flexion torque [127, 130, 143–145], it is difficult to determine whether some of this effect is explicable by an increased muscle activation capacity. Studies have differed in the muscle activity patterns observed during maximum knee flexion strength tests performed at different hip flexion angles, as assessed using EMG amplitudes [127, 130,143–147] (see Table 3). There is a trend toward a lower hamstring activity with increased hip flexion up to 90° [144, 147] but in most cases this is small and not statistically significant [127, 130, 143–145]. Angles greater than 90° have been rarely examined, although a decrease in surface BFlh EMG from 90° to 135° hip flexion has been reported [143]. Nonetheless, in addition to several important methodological limitations relating to the use of surface EMG methodologies, which are discussed below, additional important methodological differences also exist between studies. First, the hip and knee angles adopted during testing vary between studies. A different joint range indicates that reported activities may correspond to different lengths. Second, studies have monitored EMG levels using different types of strength tests, including isometric, isokinetic concentric or eccentric, and Nordic exercise tests. Force and EMG activity differ between contraction types and this difference may be length dependent. Third, studies have used variously either raw or normalized EMG values and this can influence the EMG-joint angle relation. Nevertheless, if the EMG data are considered to at least partially reflect muscle activation-joint angle patterns, it appears that changes in hip flexion angle do not substantially influence hamstring muscle activation. Hence, the increase in peak knee flexion torque at greater hip flexion angles [127, 130, 143–145] may be less explained by alterations in the magnitude of muscle activation and thus more explicable by anatomical (morphological) factors.

**Table 3:** Methodological details and main findings of studies that examined the influence of hip and knee joint angle on muscle activities of the hamstrings during knee flexion tests (hip neutral position = 0° in prone, unless stated otherwise, knee full extension = 0°)

	Participants		Normalization	Contraction			Angular position differences in EMG		
Study	(n, Sex)	EMG Type	(hip, knee angle)	(angular velocity, °/s)	Hip (°)	Knee (°)	BFlh	ST	SM
*Hip angle effects (greater angle–longer length)*									
Mohamed et al. [127]	19F	IM	MMT	ISOM	0, 90	90, 45, 0	NS	NS	0 < 90
Guex et al. [144]	10 (5 M-5F) sprinters	S	Raw	ISOM	0,30,60,90	45	30 > 90	NS	
Lunnen et al. [143]	16 (12F-4 M) PE students	S	Raw	ISOM	0 (supine), 45,90,135	60	0 > 135		
Worrell et al. [130]	50 (25F, 25 M)	S	Max at any angle	ISOM	0	0,30,60,90	NS		
Kellis et al. [145]	20 (10F-10 M)	S	MVC (0, 45)	CON, ECC 60,150	0,45,90	0–90	NS	NS	
Guex et al. [144]	10 (5 M-5F) sprinters	S	Raw	CON, ECC 60,150	0,30,60,90	90–0	NS	NS	-
Sarabon et al. [147]	18 (13 M-5F) active	S	MVC (0, 90)	NHE	0,25,50,75	20–90	0 > all angles	0 > all angles	
Hegyi et al. [146]	13 amateur athletes	HD	MVC (0, 30)	NHE	0,90	90–15	0 > 90	0 > 90	
Black et al. [148]	24 (12F, 12 M)	S	Raw	CON30	10 (Supine), 80	N/A		NS	
*Knee angle effects (smaller angle- greater length)*									
Mohamed et al. [127]		IM	As above	ISOM	0, 90	90, 45, 0	NS	Hip0: NS Hip90: 90, 45 > 0	NS
Kirk and Rice [149]	11 M	S	EMG at peak torque	ISOM	0	20, 90	90 > 20 at 50% MVC		
Kirk and Rice [149]	11 M	IM		ISOM	0	20, 90	90 > 20 at 50 and 100% MVC		
Onishi et al. [128]	10 M	IM	MVC (90,90)	ISOM	0	60, 90	90 > 60	90 > 60	90 > 60
Kellis and Katis [150]	9 M	S	Raw	ISOM	90	0,45,90	90 < 45,0	90 < 45 > 0	
Kumazaki et al. [79]	10 F-M	S	Raw	ISOM	0	0, 30, 60, 90	90, 30, 60 < 0	NS	90 > 60,30, 0
Worrell et al. [130]	50 (25F, 25 M)	S	Max at any angle	ISOM	0, 30, 60, 90	0, 30, 60, 90	90 < 30–60 > 0 (“hamstrings”)		
Read et al. [151]	10 M soccer players	S	MVC (0, 0)	ISOM	0 (Supine)	30, 90	90 < 30	NS	
Marchetti et al. [152]	15 M resistance trained	S	Raw	ISOM	15	0, 90	NS		
Avrillon et al. [125]	15 M (jumpers–sprinters)	S	Raw	ISOM, submaximal	90	45			
Chapman et al. [92]	10 M	S	Raw	ISOM	0–10	30,60	NS	NS	
Kellis and Baltzopoulos [153]	12F	S	MVC (90, 35)	CON, ECC	90	0–90	30–40		
Beyer et al. [154]	20 M (Sport science students)	S	MVC (0, 90)	ISOM	0	90, 75, 60, 45, 30, 15	90 < 30	NS	
Kawama et al. [155]	16 M (active)	S	Max at any angle	ISOM	0	30,60,90	NS	90 < 60, 30	90 < 60, 30
Motomura et al. [156]	20 M	S	Max at same angle	ISOM, submaximal	45	10 80	80 > 10	80 > 10	
Hirose and Tsuruike [132]	16 M (active)	S	MVC (90, 90)	ISOM submaximal	0	30, 60, 90, 120	120, 90 < 60, 30	120 > all angles 90 > 60,30	120, 90 < 60, 30
Hirose and Tsuruike [132]	16 M (active)	S	MVC (90, 90)	ISOM bridge submaximal	0	30, 60, 90, 120	120,90 < 60, 30	120, 90 < 60,30	120, 90 < 60, 30
Andriacchi et al. [157]	4 M	S	Max at any trial	Isotonic	0 (Supine)	40–0	40 > 0	40 > 0	40 > 0
Onishi et al. [128]	10 M	ITRM	EMG between 75° and 90°	CON30	0	120–0	120 to 0 ↑	120 to 0: ↓	120 to 0: ↓
Higashihara et al. [158]	10 M	S	Max at any trial	ECC10, 60, 180, 300	0	90–0	90–16 < 0–15	NS	NS
Croce and Miller [159]	13 M	S		CON 100 to 400	0	0–15, 25–40 55–70, 75–90	Middle ROM greater than end ROMs *		
Boyer et al. [160]	18 (10 M, 8F) active	S	Max (K90)	NHE	0	90–0	70–80% of motion		
Boyer et al. [160]	18 (10 M, 8F) active	S	Max (K90)	Stiff-leg deadlift	0–90		70–80% of motion		
Hegyi et al. [146]	13 amateur athletes	HD		NHE	0,90	90–15	90 to ~ 30: ↑	90 to ~ 30: ↑	
Monajati et al. [161]	10F soccer players	S	MVC (0, 30)	NHE Ball leg curls		60–0	60 to 0 ↓	60 to 0 ↓	
*Combinations of hip and knee angle effects*									
Mohamed et al. [127]	19F	IM	MMT	ISOM	0, 90	90, 45, 0	NS	H0-K90 > H90-K0	NS
*Other conditions*									
Keerasomboon et al. [162]	22 M (active)	S	MVC (0 or 30)	ISOM, CON, ECC with 5-kg load	0, 45	0, 45, 90	Hip extension superimposed to knee flexion increased EMG compared to hip extension alone 90, 45 > 0		
Hegyi et al. [163]	21 M	HD	Raw	ISOM	0	30	Hip extension superimposed to knee flexion increased EMG compared to knee flexion alone		
Hirose et al. [164]	20 M	S	MVC (H0, K30, 60, 90)	ISOM NHE	~ 0–15	30–0 50–0 90–0	90–0 > 50–0, 30–0		

*BFlh* Biceps femoris long head, *ST* Semitendinosus, *SM* Semimembranosus, *M* Males, *F* Females, *HD* High Density, *S* Surface, *IM* Intramuscular, *HD* High density, *Norm* Normalization, *MVC* Maximum voluntary contraction, *H* hip angle, *K* knee angle, *NS* non-significant differences, *ISOM* isometric, *Isok* isokinetic, *ECC* Eccentric, *CON* Concentric, *NHE* Nordic exercise

#### Knee flexion contractions—knee angle effects

Information regarding the effects of knee joint angle on hamstrings activity during knee flexion contractions varies between studies [79, 127, 128, 149–151, 158] (Table 3). In particular, BFlh EMG amplitude, measured using bipolar surface or intramuscular electrodes approximately in the middle of the muscle belly, has been reported to increase [79, 128, 132, 137, 150, 151, 154], decrease [149, 156, 158, 161], increase and then decrease [130, 157, 159] or remain unaltered [127, 152, 155] as the knee approaches full extension (longer muscle length). Similarly, ST EMG amplitude has been reported to increase [137, 155], decrease [128, 132, 149], increase and then decrease [165], or remain unaltered [79, 127, 151, 154] as the knee extends, and SM EMG signal amplitude was found to increase [79, 132, 155], decrease [128, 149], increase and then decrease [157], or remain unaltered [127, 151, 158] from flexion to full extension. Finally, BFsh activity was found to decrease by 30–50% as the knee approached full extension [79, 127], although this decrease was not always statistically significant [79]. In a recent study, decreases in both the surface EMG amplitudes and intramuscular motor unit firing rates of ST, SM and BFlh were observed at longer lengths (extended knee angle), suggesting that EMG amplitudes might at least partly reflect firing rates of the underlying motor units and that these firing rates may be slower at longer muscle lengths [149]. However, joint angle differences disappear at very low contraction intensities (< 25% of maximum)[149].

To provide a more representative view of the reviewed evidence, we examined the range of motion at which peak EMG was observed and then assigned each study to one of four categories of knee flexion angle range: small (0–30°), middle (31–60°), great (> 60°), and “no change”. The results are presented in Table 4. A great variability exists in EMG-knee angle patterns between studies, which can be attributed to various factors such as the type of test and EMG processing and analysis methods adopted (see further below). Further, most studies comparing EMG between knee angles kept the hip in the neutral position (Table 3) whilst the knee was (generally) moved through 90°,and rarely 120° [128, 132]. Τhis corresponds to muscle lengths spanning the end of the plateau region and the descending limb of the force-length relation (Fig. 4), and hence, these results reflect changes across only a limited operating length range of the hamstrings. Within these limitations, evidence from EMG studies indicate that maximum hamstring EMG is achieved in mid-range knee flexion angles during knee flexion contractions with a fixed hip angle. In this operating range, predicted active force is greater when the knee angle is 90° while passive forces are essentially absent (Fig. 5), suggesting that the peak EMG activity and predicted muscle *force* operative range do not coincide. The area of peak EMG amplitudes, however, occurs within the area of peak *torque* development [20, 128, 153, 158], even though it does not appear to be a major factor influencing it (i.e. muscle force appears to be underpinned by muscle architecture, and the torque-angle relation is then impacted by muscle moment arms).

**Table 4:** Classification of studies based on the knee flexion range of motion at which greater EMG was observed during knee flexion contractions. Studies have been classified in four categories: 0–30°, 31–60°, > 60° and those that reported no change in EMG. (*ISOM*  Isometric, *ISOK*  isokinetic exercise, *ISOT*  constant load exercise, *NHE*  Nordic exercise, *SDL*  Stiff leg deadlift)

	Range of motion of peak EMG (°)			
	0–30	31–60	> 60	No change
BFLH	Isometric			
	Kellis and Katis [150] Kumazaki et al. [79] Read et al. [151]	Worrell et al. [130] Hirose and Tsuruike [132] Hegyi et al. [146]	Kirk and Rice [149] Onishi et al. [128] Motomura et al. [156]	Kawama et al. [155] Mohamed et al. [127] Marchetti et al. [152] Chapman et al. [92]
	Dynamic			
	Onishi et al. [128] (ISOK) Beyer et al. [154] (NHE, SLD)	Croce and Miller [159] (ISOK) Andriacchi et al. [157] (ISOT)	Higashihara et al. [158] (ISOK) Monajati et al. [161] (NHE)	
ST	Isometric			
		Mohamed et al. [127] Kelis and Katis [150] Worrell et al. [130] Kawama et al. [155] Hirose and Tsuruike [132] Hegyi et al. [146]	Kirk and Rice [149] Onishi et al. [128] Motomura et al. [156] Hirose and Tsuruike [132] Monajati et al. [161]	Kumazaki et al. [79] Read et al. [151] Marchetti et al. [152] Chapman et al. [92]
	Dynamic			
		Andriacchi et al. [157] (ISOT) Croce and Miller [159] (ISOK)	Onishi et al. [128] (ISOK)	Higashihara et al. [158] (ISOK) Beyer et al. [154] (NHE; SDL)
SM	Isometric			
		Worrell et al. [130] Kawama et al. [155] Hirose and Tsuruike [132]	Kirk and Rice [149] Kumazaki et al. [79] Onishi et al. [128]	Mohamed et al. [127]
	Dynamic			
		Andriacchi et al. [157] (ISOT)	Onishi et al. [128] (ISOK)	Higashihara et al. [158] (ISOK)

#### Hip and knee angle effects—knee flexion contractions

By manipulating only one joint whilst keeping the second joint fixed, most studies have examined only specific regions of the hamstring operating length range. This does not allow a full picture of the relation between muscle length, force, and activation to be developed. To the best of our knowledge, only one study has presented intramuscular EMG data in various hip and knee joint positions and, hence, a wider range of muscle lengths [127]. Length-dependent variations in EMG were found between muscles, but these were small and inconsistent. Peak knee flexion torque markedly increased and peak EMG activity tended to decrease from shorter (hip flexion 90°–knee flexion 0°) to longer (hip flexion 0°—knee flexion 0°) lengths [127]. The influence of length on muscle (EMG) activity is still controversial, not only for the hamstrings but also for other muscles [143, 166–169]. The reported findings for the hamstring muscles tend to support the notion that the activation-muscle length relation does not coincide with the torque/force-length relation. The maintenance or decrease in activation at longer lengths coinciding with an increase in peak force/torque suggests that contribution of muscle activation to peak torque development is probably minimal when compared to other factors such as passive/elastic force, muscle length (when the test is performed at intermediate length range) or moment arm, at least in the populations studied, but the precise influence of each factor may differ between muscles. By contrast, the greater activation at shorter lengths may indicate that neuromuscular activation may serve to increase muscle force/torque development in parts of the force-length relation at which active forces are low, partially overcoming the apparent mechanical limitation.

#### Differences between muscles

Research has also examined length-dependent hamstrings EMG activities differences between muscles. Studies that have compared EMG activities between knee flexion angles with the hip in a fixed position have shown that the angle of peak EMG amplitude differs between muscles, occurring at 25–30° of knee flexion in BFlh [79, 127, 132, 145, 151, 154] but 40–50° [128, 132, 145, 159] or even 90–105° [127, 128] in ST. In SM, the angle of peak EMG amplitude was found to occur at 30–60° of knee flexion [127, 132, 155] or greater angles [79, 128]. There are, however, studies reporting BFlh [158], ST [79, 151, 154, 155, 158] and SM [158] activities being consistent across knee joint angles.

Again, methodological variations in EMG recording and analysis and testing protocols between studies (such as contraction type and intensity, range of motion, test position) and an inherent variability in the EMG signal have an important effect on the angle of peak EMG amplitude. However, even studies using similar protocols report different results. Three studies, for example, examined muscle activity during isometric contractions at the same knee joint angles and using similar (intramuscular) EMG recording methodology. Mohamed et al. [127] found that the peak EMG amplitude does not differ between knee joint angles in BFlh and SM, but it is lower at 0° than 45 and 90° (with 90 hip angle) in ST (Table 3). Onishi et al. [128] found BFlh and SM peaks to occur near full extension (in contrast to Mohamed et al. [127]) whilst ST EMG occurred in greater knee flexion (in agreement with Mohamed et al. [127]). Finally, a greater surface EMG amplitude but lower (intramuscular) motor unit firing rate in BFlh than ST and SM was observed during isometric contractions at 50% οf maximum joint torque [149]. Interestingly these intermuscular differences were not length dependent (as determined by changes in knee angle) [149]. The picture provided by these three studies illustrates that, even when the testing methodology and conditions appear similar, the resulting EMG-length relations may differ considerably between studies.

As seen in Table 4, BFlh EMG can occur anywhere within the range of motion; nevertheless, it could be suggested that BFlh shows greater activity at shorter muscle lengths than ST and SM. In addition, ST and SM activity tends to peak in the mid-range or towards greater knee flexion. Avrillon et al. [125] found that the product of PCSA, surface EMG and moment arm of ST was lower than for BF (including BFlh and BFsh) and SM during submaximal isometric tests performed from 90° hip angle and 45° knee angle. However, they did not find differences in activity between the hamstring muscles and, therefore, it is still unclear whether torque production at specific knee angles evokes a selective activation of specific hamstring muscles. In the same study the authors [125] noticed large individual differences in EMG activity of each hamstring relative to the other muscles, which were considered as evidence of individual-specific muscle activation strategies.

Using the average EMG amplitude during exercise, researchers have asked whether specific exercises might preferentially recruit a particular hamstring component [19, 29, 170, 171]. Taking the average amplitude of EMG signal may be considered as an index of muscle recruitment, but it does not provide information on the activation-length relations. Nevertheless, a recent systematic review concluded that barbell hip thrust, which can be considered as a hip dominant exercise, promotes greater BFlh than ST EMG activity [172]. However, another review reported a large variability in the reported findings and thus concluded that differentiation of exercises based on EMG amplitude is problematic [19]. Based on a review of fMRI studies, the same authors [19] concluded that knee dominant exercises such as Nordic or leg curl exercises selectively recruit ST whereas hip dominant exercises such as stiff-leg deadlifts appear to preferentially activate BFlh and SM [19]. This was attributed to the greater BFlh moment arm, and hence torque generation, at the hip than at the knee in these exercises (Table 3) [19]. Additional factors, however, are likely to contribute to these results, such as the bi-articular function of the hamstrings, their potential recruitment in rotation movements [173–176], and the influence of movement velocity on force and activation. However, examination of these factors and their complex interactions during various exercises is beyond the scope of this review.

Another question that has attracted some attention is whether the two BF heads display different activation patterns. BFsh and BFlh are innervated by different nerve branches; BFlh by the tibial portion of the sciatic nerve and BFlh by the common peroneal branch of the sciatic nerve [177]. Differential BFsh and BFlh innervation has been proposed to potentially result in distinct activity patterns and explain the predominance of BFlh over BFsh injury [178]. This, however, remains unsubstantiated, mainly because the anatomical arrangement of the two muscles prevents accurate surface EMG measurements being obtained from each muscle. Nevertheless, studies comparing activity between the two heads using intramuscular [127, 128] or surface [79] electrodes reported that BFsh may be less activated than BFlh near full knee extension. Thus, BFlh may compensate for BFsh at extended knee positions. Studies using functional magnetic resonance imaging (fMRI) also support a differential recruitment between BFlh and BFsh [18, 179–181]. Yanagisawa and Fukutani [179], for example, reported greater BFsh than BFlh recruitment during knee flexion contractions with the hip in extension (which corresponds to relative shorter lengths) but there were no differences between these two muscles when the hip was flexed, suggesting that recruitment differences during maximum knee flexion efforts depend primarily on hip joint position. Other studies have reported greater BFsh than BFlh recruitment during eccentric leg curls [181], inertial flywheel leg curls [182], and Nordic exercises [18, 180], which also tend to be performed with the hip in extension, while hip extension exercise resulted in a greater BFlh than BFsh recruitment [18]. It is not known whether these differences are associated with BFlh injury risk or whether they are influenced by training status or fatigue.

#### Influence of testing conditions

The activation-joint angle relation may also differ between eccentric and concentric contractions [22]. Lower EMG amplitudes during eccentric contraction are often attributed to neural inhibition [158] as part of a modified neural strategy that is initiated at both supraspinal and spinal levels [183]. However, few studies have compared the two contraction types in the hamstrings and these studies have shown no systematic differences in EMG-joint angle patterns between contraction modes [144, 145]. This is in line with a recent review concluding that it is unclear whether activation differences between muscles or exercises, which are reported in the literature, are due to differences in contraction type alone [19].

It has also been suggested that neural activation may influence the torque-angle relation [22]. At the commencement of a contraction, a greater neural activation increases the rate of force or torque development, shifting the peak torque measured during a concentric contraction toward longer muscle lengths (i.e. earlier in the movement) [184]. Consequently, it was suggested that the optimum angle for torque production can occur without the influence of the mechanical properties of the muscles being tested [22]. By contrast, neural inhibition has been observed at selected parts of the range of motion [24] or during eccentric tests [158] and this may reduce recorded EMG activation, especially at longer lengths.

#### Methodological considerations

The great variability in reported EMG activation patterns between studies raises concerns about making generalized conclusions regarding length-dependent variations in hamstring activation. As explained previously, an important source of variability is the difference in hip and joint ranges of motion between studies, despite few experiments testing angles greater than 90° (Table 3). In addition, lateral tibial rotation may occur at terminal knee extension and should theoretically decrease BFlh length and increase SM and ST lengths [154, 158]. This phenomenon, however, needs further verification. Second, recording of the EMG signal of each of the hamstring muscles using surface electrodes is methodologically difficult as some muscle bellies overlap, and therefore cross-talk between signals is likely to be high [185]. Studies using intramuscular techniques overcome this limitation [127, 128, 149] yet the reported results are still conflicting, possibly because fewer motor units are studied and the output received by electrodes may be less representative of the whole muscle (when compared to surface EMG acquisition). Surface EMG signals are also influenced by muscle movement of the muscle relative to the electrodes, which is more evident during dynamic joint movements but still occurs during “isometric” (fixed end) contractions due to stretch of the series elastic component [186]. Electrode proximity to a tendon or innervation zone may result in reduced EMG signal amplitude and this may vary between contraction levels and muscle lengths [186, 187]. Hence, interference in signals between adjacent muscles in combination with differences in the location of the bipolar surface electrodes might have contributed to the notable differences in recorded EMG-knee joint angle effects between studies.

Third, a variety of methods have been used to analyze the EMG signal (Tables 2 and 3). Specifically, length-dependent variations have been assessed using both raw [79, 125, 143, 144, 150, 152, 163] and normalized [127, 128, 130, 132, 137, 145–147, 149, 151, 154–157, 162, 164] EMG values (Tables 2 and 3). Raw EMG values show higher individual variability than normalized EMG values, but they allow direct comparison between different joint angle conditions. EMG signal normalization reduces individual variability but it is highly dependent on the type of test or the method of obtaining the reference value. Many researchers have used a reference value obtained during MVC [127, 128, 130, 132, 137, 145–147, 149, 151, 154–157, 162, 164]. This tends to be the recommended standard because it is reliable and easier to interpret [187] although it is problematic to then normalize EMG obtained at one angle to the EMG obtained during MVC at another angle. This is probably why some studies have used the maximum value obtained during any angle as a reference value [130, 155, 156]. Further, as seen in Tables 2 and 3, the MVC testing position varies between studies, so it is difficult to compare EMG amplitude results between studies. Alternative techniques have also been implemented, including to express EMG amplitudes as a percentage of EMG recorded during a particular range of the motion (75–90° of knee flexion) [128] or during a series of dynamic isokinetic tests [158]. This enables a better comparison of EMG values between different phases of the movement, but it makes comparisons between muscles more difficult and does not circumvent the problem of movement of the muscle(s) beneath the electrodes. Finally, others have used a value obtained during a knee flexion movement combined with medial (for SM, ST) or lateral (for BFlh) rotation against manual resistance provided by the experimenter [127]. This latter technique assumes that maximum EMG is observed when knee flexion is combined with medial rotation or lateral rotation for the SM/ST or BFlh, respectively. Hence, it differs significantly from other procedures used in the aforementioned studies. As the test used to obtain the EMG normalization values varies between studies is not the same for all muscles, and it is uncertain whether subjects exert maximum effort against the resistance provided manually by the experimenter, this normalization method may not be ideal and makes between-study comparisons relatively difficult.

It is certain that the EMG collected from one component cannot be considered as representative of whole hamstring muscle group. Intramuscular electromyography is most adequate for studying the hamstrings, especially when attempting to examine activation of specific neuromuscular compartments, although high-density surface EMG arrays may be of increasing use in future experiments in order to detect motor unit firing patterns using a surface EMG strategy. Perhaps the combination of intramuscular/high-density electrodes and diagnostic imaging techniques may provide a more precise tool for correct identification and study of hamstring muscle activation.

#### Implications

There is inconsistent evidence regarding length-activation relations of each hamstring muscle as well as differences in length-activation patterns between hamstring muscles. Methodological difficulties related mainly to use of EMG measurements but also the complicated anatomy of the muscle group may account for these significant between-study variations. Performing knee flexion or hip extension contractions from greater hip flexion angles tends to decrease recorded EMG activity. During knee flexion contractions with the hip angle fixed, the range of motion at which peak muscle activity is observed varies between muscles. A tendency exists for a greater ST, SM and BFsh activation in 90–30° of knee flexion while maximal BFlh activation could be observed anywhere in the range of motion, including shorter knee angles (0–30°). BFsh activity tends to be greater during knee flexion contractions with the hip in extension, and thus BFlh may be preferentially activated when the hip flexes or the knee extends. With the caveat that there are several identified limitations of EMG-based techniques, the current evidence suggests that hamstring muscle length-activation relation is not similar to the force-length relation, so activation may only play a small role in most conditions. Further, caution is advised in accepting the assumption that the higher EMG amplitudes in mid-range angles might directly account for the larger joint torque in this region since this EMG peak does not align with the greatest muscle force.

### Torque-angle relationship

The force-length and moment arm-angle relations combine to produce a torque-angle relationship, which dictates our performances across tasks. In voluntary contractions, the knee flexion torque-joint angle relationship is formed by plotting the isometric torque obtained across joint angles or by recording torque during a dynamic contraction. Table 5 shows angle of peak torque values in various testing conditions, as reported in the literature. The majority of included studies support that the maximum hip extension [130, 188–192] and knee flexion [80, 127, 143–145, 148, 193–197] strength increases as the hip is flexed. This can be attributed to the greater hip and knee hamstrings force (Fig. 4) and hip moment arm (Table 1) as the hip flexes. Irrespective of hip flexion angle, the maximum knee flexion torque is centered around 30° of knee flexion (Table 5) and ranges between 0 and 45° of knee flexion during isometric [79, 127, 130, 145, 149, 150, 152–155, 158, 198–202] and 15–70° during isokinetic [20, 24, 128, 153, 158, 192, 203–221] tests.

**Table 5:** Methodological details and main findings of studies examining the influence of hip and knee joint angle on hamstrings torque (hip neutral position = 0°, knee. full extension = 0°). * Angle of peak torque is based on visual inspection or no statistical comparison between angles is mentioned. Empty cells indicate that information was not provided

Study	Participants (n, Sex)	Hip angle (°)	Knee angle (°)	Type of test (Angular velocity in °/s)	Angle of peak torque
*Knee angle effects on knee flexion torque*					Knee angle
Murray et al. [198]	48 M	Seated	30, 45, 60	ISOM	30, 45 > 60
Nikose et al. [199]	50 with ACL reconstruction surgery	0 (Prone)	0, 30, 45, 90, 105	ISOM	30
Ullrich et al. [200]	32 (23 M, 9F) athletes	0 (Prone)	30, 40, 50, 60, 70, 80, 90	ISOM	29.2
Balle et al. [222]	20 M	90	90, 70, 50, 30	ISOM	70
Alonso et al. [223]	20 (10F, 10 M)	40	89, 76, 63, 50, 37	ISOM	63
Nomura et al. [201]	24 (10 M, 10F)	0	30, 45, 60, 90, 105	ISOM	30*
Nara et al. [202]	28 m	85	30, 60, 90	ISOM	30 > 60 > 90
Onishi et al. [128]	10 M	0 (Prone)	60, 90	ISOM	15–30
Kellis and Katis [150]	9 M	90 (Seated)	0, 45, 90	ISOM	0 > 45,90
Kumazaki et al. [79]	10 F-M	0 (prone)	0, 30, 60, 90	ISOM	0 > 30,60,90
Kirk and Rice [149]	11 M	0 (prone)	20, 90	ISOM	20 > 90
Marchetti et al. [152]	15 M resistance trained	15	0, 90	ISOM	0 > 90
Beyer et al. [154]	20 M	0	90, 75, 60, 45, 30, 15	ISOM	30
Chapman et al. [92]	10 M	0–10 (prone)	30, 60	ISOM	NS
Kawama et al. [155]	16 M	0 (prone)	30, 60, 90	ISOM	30 > 60,90
Onishi et al. [128]	10 M	0 (prone)	0–90	CON30	15–30
Read et al. [203]	27 M soccer players	90		CON60	31 ± 8
Mikami et al. [204]	30 M	-		ECC60, 300	ECC60: 10–30 ECC300: 20–30
Moltubakk et al. [205]	22F elite rhythmic gymnasts 16 F other sports	90	0–90	CON60	40 ± 13 (gymnasts) 57 ± 20 (others)
Ogborn et al. [206]	18F, 14 M	0 90	5–95	CON90	39.4 ± 9.7 31.6 ± 7.4
Brughelli et al. [207]	18 M cyclists, Austrialian rules football players (AFP)	90	0–110	CON60	26.2 ± 2.9 (Cyclists) 32.3 ± 3.8 (AFP)
Brockett et al. [208]	10 (8 M, 2 F)	90	0–90	CON60	38
Brockett et al. [209]	23 M- Injured 18 M athletes, non-injured athletes	90	0–110	CON60	30.1 ± 1.5 (Uninjured) 40.9 ± 2.7 (Injured)
Brughelli et al. [210]	24 M soccer players	90	0–110	CON60	30.4 ± 2.7 to 32.2 ± 3.6
Maciel et al. [211]	189 M soccer players	N/A	5–95	CON60, 240	31.28 ± 8.67 to 37.92 ± 10.23
Kannus [212]	21 (9 M, 12F) with injuries	Seated	0–90	CON60, 180	CON 60:38 ± 8.6 CON180: 40.5 ± 7.0
Kannus and Beynnon [213]	249 (106F–143 M)	100	0–90	CON60	33 ± 8.0 (M) 37 ± 10.0 (F)
Kannus and Beynnon [213]	249 (106F–143 M)	100	0–90	CON180	40 ± 10.0 (M) 44 ± 11.0 (F)
Baumgart et al. [214]	2-(10F-10 M) athletes	10,90	10–90	CON60	H90 > 10 H10: 38.1 ± 13.2 H90: 26.9 ± 8.9
Worrell et al. [130]	50 (25F, 25 M)	0 (prone)	0, 30, 60, 90	ISOM	0,30 > 60,90
Baumgart et al. [214]	2-(10F-10 M) athletes	10,90	10–90	CON180	H10: 68.5 ± 6.9 H90: 61.2 ± 11.5
Pieters et al. [192]	116 M football players	Seated	0–100	CON60, 240	30 *
Sole et al. [24]	15	Seated	0–90	CON60, ECC60	CON60: 85–26 > 25–5 ECC60: 5–45 > 46–85
Sousa et al. [215]	30 M basketball players	85	0–90	CON60, ECC60	CON60:30–60 ECC60: 55
Cohen et al. [216]	9 M soccer players	Seated		CON120, ECC120	CON120 = 30 ECC120 = 10
Kellis and Baltzopoulos [153]	12F	Seated	0–90	CON30, 60, 90, 120, 150 ECC30, 60, 90, 120, 150	30–40
Correia et al. [224]	12 M football players	85	0–90	CON60 CON180 ECC60 ECC180	23.4 ± 8.1 36.3 ± 12.4 18.1 ± 13.2 19.4 ± 10.8
Çınar-Medeni et al. [217]	27 M	90	30–90	CON, ECC60	40
Çınar-Medeni et al. [217]	27 M	90	30–90	ECC60	44
Huang et al. [225]	46 M	85	20–90	CON60, ECC60	CON60:40–60 ECC60: 50–70
Delextrat et al. [218]	25F hockey players	90	0–90	ECC120	10–40*
Eustace et al. [219]	34 M soccer players	90	-	ECC60, 180, 270	40 > 70*
Nishida et al. [220]	6 M	0	0–90	ECC60	24.1 ± 10
Page and Greig [221]	13 M soccer players	90		ECC60,300	ECC60: 32 ± 9 ECC300:46 ± 14
Baumgart et al. [214]	2-(10F-10 M) athletes	10,90	10–90	ECC60	HA10:36.1 ± 15.2 HA90: 32.4 ± 16.0
Higashihara et al. [158]	10 M	0 (Prone)	90–0	ECC (4 speeds)	15–30
*Hip angle effects on knee flexion torque*					Hip angle
Mohamed et al. [127]	19F	0, 90	90, 45, 0	ISOM	90 > 0
Guex et al. [144]	10 (5 M-5F) sprinters	0,30,60,90	45	ISOM	90 > 60, 30, 0
Lunnen et al. [143]	16 (12F-4 M) PE students	0 (supine), 45,90,135	60	ISOM	135 > 90,45,0
Ogborn et al. [194]	44 (22F, 22 M)	0 (Supine), 90	90	ISOM	90 > 0
Bohannon et al. [197]	19 (10F-9 M)	0,90, 120	90	ISOM	120 > 90 > 0
Bohannon et al. [196]	12 Hemiparetic patients	0,95	90	ISOM	95 > 0
Kellis et al. [145]	20 (10F-10 M)	0, 45, 90	0–90	CON60, 120, 150	90, 45 > 0
Guex et al. [144]	10 (5 M-5F) sprinters	0, 30, 60, 90	0–90	CON – ECC 60–150	90 > 0
Black et al. [148]	24 (12F, 12 M)	10 (Supine), 80	N/A	CON30	80 > 0
Findley et al. [226]	10 (6F, 4 M)	0 (Prone), 110	0–90	CON60, 120, 180, 240, 360	NS
Bohannon et al. [195]	14F	30,95	0–90	CON60	95 > 35
Hopkins et al. [193]	14 (7F, 7 M)	10, 110	N/A	CON60, 180	110 > 10
*Hip effects on hip extension torque*					Hip Angle
Cahalan et al. [188]	72 (37F, 35 M)	45, 90	90	ISOM	90 > 45
Worrell et al. [130]	50 (25F, 25 M)	0, 30, 60, 90	90	ISOM	90 > 60, 30, 0
Kindel and Challis [189]	21 (11F, 10 M)	45, 30, 15, 0 (Prone)	0,90	ISOM	45 > 30, 15, 0 30 > 15, 0
Goodwin and Bull [227]	10 M	0, 20, 30, 40, 50 (supine)	Angle changed in each position	ISOM (Hip Thrust)	NS
Bertoli et al. [228]	17F	15, 60, 90, 100	Flexed	ISOM	100 > 90 > 60, 15 60 > 15
Kindel and Challis [190]	18 (16F, 2 M)	45, 30, 15, 0 (Prone)	0, 90	ISOM	45 > 30, 15, 0 30 > 15, 0
Bazett-Jones et al. [191]	29F	0, 30, 90 (Prone)	90	ISOM	90 > 30 > 15
Pieters et al. [192]	116 M football players	0–90 (supine)	0	CON60, 240	60*
*Knee angle effects on hip extension torque*					Knee angle
Kindel and Challis [190]	18 (16F, 2 M)	45, 30, 15, 0 (Prone)	0, 90	ISOM	0 > 45
Kindel and Challis [189]	21 (11F, 10 M)	45, 30, 15, 0 (Prone)	0, 90	ISOM	0 > 45
Kwon and Lee [137]	20 M & F	0 (Prone)	0, 30, 60, 90, 110	ISOM	0 > 90, 60, 30, 0

*M*  Males, *F*  Females, *ISO*  Isometric, *CON*  Concentric, *ECC*  Eccentric, *HA*  Hip flexion angle, *KA*  Knee angle

Several studies have also shown that the maximum knee flexion torque occurred at more flexed knee angles when the hip was more flexed [127, 144, 145, 206] which makes sense given that this would approximately maintain muscle length; i.e., the muscle length rather than the joint angles themselves appear to dictate muscle strength. For example, peak knee flexion torque was observed at 0° of knee flexion when the test was performed at 0° hip angle but shifted to ~ 45° when the test was performed with a 90° hip angle [127, 145]. Thus, the optimum knee flexion angle shifts toward flexion as the hip is flexed in a strength test.

**Fig. 8:**
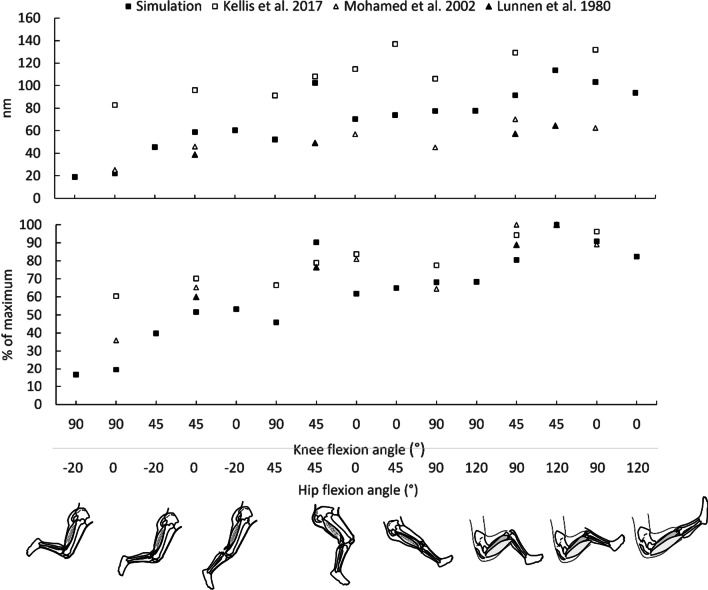
Absolute (upper graph) and relative (lower graph) peak knee flexion torque values reported in studies examining muscle strength at various combinations of hip (H) and knee (K) flexion angles. The average predicted torque resulting from forward simulation using five different models is also included for comparison. Joint positions are arranged, from left to right, from shorter to longer muscle lengths. For each data set, torque values are expressed relative to the peak value to allow better comparison between studies (0° = neutral hip position, negative hip angle indicates extension; 0° = full knee extension)

To appreciate the relation between experimentally recorded torque-angle data and the force-length curve of the hamstrings, torque should be measured with various combinations of hip and knee joint angles and then the data plotted with angular positions arranged from shorter to longer lengths. A few experimental studies have provided such information [127, 145]. As shown in Fig. 8 [127, 145], the lowest recorded isometric torque is achieved when the hip is in the neutral position (0°) and the knee flexed at least to 90° whilst the greatest value is observed when the hip is flexed to 90° or 120° with the knee angle ≤ 45° [127, 145]. These results are consistent with our predictions using mathematical simulation, which additionally show that torque capacity is lower when the hip extends beyond 0° and knee flexes past 90° (shorter lengths) and increases when hip flexion is 120° and knee angle is 45° (longer lengths). Torque then decreases at even longer lengths, as the hip angle exceeds 90° and the knee is fully extended. By comparing the experimentally recorded knee flexion torque-angle data (Fig. 8) to our torque-angle (Fig. 7) and force-length simulation results (Fig. 4), bi-articular hamstrings are found to generate maximum isometric knee flexion force at a hip flexion angle of 45°-90° while peak knee flexion torque occurs at longer lengths, between 90° and 120° hip angles.

Knee flexion torque is generally greater during isokinetic eccentric than concentric strength tests (for reviews see [183, 229]). Based on the reviewed evidence (Table 5), most studies have reported that the angle of peak torque during eccentric tests is in the range 30–40° flexion, which is similar to that observed during concentric contractions. Further, most studies that examined either the shape of torque-joint angle relation or angle of peak torque have reported no differences between the two contraction types [153, 215, 216, 225, 230]**.** Only two studies provided evidence that peak concentric isokinetic torque occurs at a greater knee flexion angle (shorter length) than peak eccentric torque [24, 153]. Hence, it appears that shape of isokinetic knee flexor torque-angle curves does not differ between the two contraction types, even though the mechanisms that contribute to force development are contraction-dependent [183].

Evaluation of torque production across muscle lengths using resistive dynamometers is not without limitations [231, 232]. A common observation reported by several authors is a considerable individual variability in the angle of peak torque during maximum flexion contractions, especially between knee flexion angles of 0 and 45° [127, 130, 145, 198]. This variability has been attributed to methodological limitations such as the level of participant motivation and the low reliability of angle of peak torque measurements [22]. Based on the reviewed evidence, the shape of the torque-angle relation may also be affected by inter-individual variability in each single factor that influences the torque-angle relationship, namely active and passive force development, moment arm, neural activation, and individual muscle architecture. Methodological limitations of isokinetic dynamometers such as the influence of gravity and inertia on torque at various joint angles have also been recognized [229, 232]. For example, during the initial and final phases of the isokinetic motion, the knee accelerates and decelerates [233]. In most cases, these data are not taken into consideration due to the influence of inertia on the recorded torque measurements. Thus, portions of joint motion near knee extension and flexion may not be represented in the torque-joint angle relationship.

#### Implications

One aim of the present review was to determine whether exercise testing protocols that have been used to screen athletes for strength deficits cover the full operating length of the hamstrings. Early studies showed that individuals with a prior hamstring injury present a shift in their angle of peak torque toward greater knee flexion angles during slow speed concentric contractions [207–209]. Recent studies, however, have presented mixed results. Some studies, for example, report that the shift in angle of peak torque after hamstring injury occurs during eccentric contractions only [24, 204], another study found this shift during isometric contractions [202], and Correia et al. [224] did not observe a shift during concentric or eccentric tests. The use of torque-joint angle relations during contractions for detecting an individual with hamstring muscle malfunction relative to a typical population has been discussed elsewhere [22]. Nevertheless, these studies used knee flexor strength testing protocols involving isometric or dynamic knee flexions in a seated position (hip angle 90°) and 90° range of knee motion. This range does not cover the full operating length of the bi-articular hamstrings but does correspond to a less optimum range for torque development (Fig. 7), whilst it is within the optimum range for force development (Fig. 4). To best of our knowledge, it is not known whether such shifts occur when strength tests are performed with different hip positions (prone or flexed hip > 90°).

Typical knee flexor strength testing protocols involve isometric or dynamic knee flexions in a seated position (hip angle 90°), and knee flexion strength tests are rarely performed with hip angles > 90° (Table 5) even though knee flexion torque may be greater with the hip more flexed. In contrast, strength tests performed with minimal or no hip flexion ~ 0° (e.g. leg curls in the prone position or Nordic exercise) will correspond to a less optimum range for knee flexion torque production (Fig. 7). Nevertheless, as shown in Table 5, typical isokinetic concentric, eccentric, or isometric strength testing would provide greater torque values in the range of 30–40° of knee flexion. Hence, if the same test is used to examine both legs, differences in torque-joint angle relations between legs would probably be strongly considered as indicative of hamstring functional alteration.

Another question that was raised in this review is whether a change in the joint torque-angle relationship reflects changes in the properties of each individual hamstring muscle. Based on our model predictions (Fig. 4) and examination of published moment arm and activation data (see previous sections), during a typical 90° knee flexion strength test from a seated position, SM impacts joint torques more when in greater knee extension but ST increases prominence when in greater flexion. BFlh tends to show greater torque at more extended angles than ST but it is mainly recruited in mid-range angles. Within the limitations of the present research, one might conclude that a shift of peak torque toward smaller knee flexion angles may reflect reductions primarily in SM and, secondarily, in BFlh contribution to torque.

Due to the influence of moment arm and activation, the optimum range for torque production occurs at longer muscle lengths (Figs. 7, 8) than the corresponding force optimum range (Fig. 4). This impacts the torque-joint angle relation but it depends on the hip joint position during the test. During leg curls, for example, the optimum ranges for force and torque development almost coincide (from 45° to 0° flexion). In resistive knee extension exercises in the seated position, torque output tends to occur at knee angles 0° to 45° even though the optimum range for force development occurs at more flexed joint angles. In hip dominant tasks, including the late lowering phase of the good morning exercise [98], where the hip flexes up to 80° and the knee is only slightly flexed, the hamstrings operate at sub-optimal lengths for hip torque generation. Exercises requiring a combination of dynamic hip flexion from 45° to 120° and knee extension from 45° to 0° may theoretically provide a more optimum exercise stimulus. This is consistent with research findings showing that training at longer lengths results in greater muscle hypertrophy than training at shorter lengths [234] and is consistent with recent recommendations for hamstring exercise selection [19].

### Limitations

In the present paper, sagittal plane forces, moment arms and joint torques were examined. Hamstring force-length relations may be altered when the sagittal plane movements are combined with movements in other planes (tibial [127] or hip rotations [173], for example). In addition, the bi-articular function of the hamstring muscles during simultaneous hip and knee joint movements and the influence of contractile velocity were not considered. Importantly, modelling data were extracted from several typical muscle-driven models for a representative male individual [Additional file show this in more detail (see Additional file 1)]. These provide an indication of force-length patterns of the hamstrings but they cannot be generalized to all individuals. Mathematical models from which data were extracted were also created to simulate walking or running, so alterations in the input data to optimize the models was only done in this context. It is possible that optimizations completed on other tasks might yield different outputs, although this has yet to be determined. In addition, force and torque predictions carry several limitations such as they are specific to cadaveric data sets that have been used as inputs, they display some errors in predicted changes in tendon slack length [50] and moment arm lengths of some muscles [51], and they may not account for short range muscle stiffness or history dependent force changes [54]. These errors may impact force predictions [235]. A greater understanding of these issues from ongoing research may allow more confidence in conclusions drawn from future analyses.

## Conclusions

We reviewed almost 100 experimental studies and used five simulation models to address five questions relating to hamstring function. With respect to the first aim, only two studies detailed the length-tension properties of sarcomeres or fibers within human hamstrings muscles. Using this information and simulation outputs, we observed that the optimal range for force production ranges from 90° hip flexion and 70°–80° knee flexion to 45° hip flexion and 10°–30° knee flexion. Owing to inter-muscular architectural differences, BFlh and SM contribute greater forces through much of the hip and knee joint ranges of motion whilst ST produces less force and has a flatter active force-length relation.

With respect to the impact of moment arm on hamstring function, the existing literature indicated that the hamstrings’ maximum moment arms are greater at the hip than knee, so the muscles tend to act more as force producers at the hip but generate greater joint rotation and angular velocity at the knee for a given muscle shortening length and speed. The long-fibered ST has a longer moment arm than SM and BFlh, partially alleviating the reduced force owing to its smaller PCSA but also reducing its otherwise substantial excursion potential. Further, owing to their shorter moment arms, SM and BF may experience better conditions for elastic energy storage-reuse than ST. Moment arm differences therefore tend to reduce functional differences between the hamstrings components that would exist according to their architectures alone.

We also examined how muscle activation impacts hamstrings torque-angle relations. Whilst there were more than 35 experimental studies that detailed “activation-length” patterns of the hamstrings, as estimated using electromyography, there is great variability in the reported findings. This variability may be due to methodological factors in relation to the data acquisition but also to variation in activation strategies used by different individuals. Within these limitations, it appears that an increase in hip flexion angle tends to decrease recorded EMG activity. During knee flexion contractions, ST, SM and BFsh tend to increase their EMG activity from mid-range to greater knee flexion angles whilst maximal BFlh activity can be observed anywhere in the range of motion, including shorter knee angles (0–30°). In most testing conditions, the hamstrings muscle length-activation relation is not synchronous with the force-length relation, so the effect of activation may only play a small role, mainly at shorter lengths. More detailed studies using advanced techniques may provide better insight into the true activation properties of the muscles and the contribution of activation to the torque-angle relation.

Maximum hip extension and knee flexion torques increase as the hip is flexed whilst the maximum knee flexion torque occurs around 30° knee flexion angles. Typical knee flexion tests involving knee flexions in a seated position and 90° range of knee motion do not cover the full operating length of the hamstrings and correspond to a less optimum range for torque development. Performing knee flexion exercises from hip angles > 90° may result in greater torque while during typical exercises from the prone or supine position the hamstrings work at a less optimal range for torque development. Owing to the influence of activation, architecture and moment arm, the optimum range for *torque* development is shifted towards longer muscle lengths (more flexed hip and extended knee) compared to the corresponding range for *force* development. Further, it can be suggested that SM impacts joint torques more when in greater knee extension but ST increases prominence when in greater flexion. BFlh tends to contribute more substantially to torque at more extended angles than ST but it is mainly recruited in mid-range angles.

During daily activities such as walking or sitting down, the hamstrings appear to operate on the ascending limbs of their force-length relations while knee flexion exercises performed with hip angles 45–90° promote more optimal force generation. Exercises requiring a combination of dynamic hip flexion from 45° to 120° and knee extension from 45° to 0° may provide a more optimum exercise stimulus if the stimulus is considered to be optimized by a high force production. Strength exercises performed at optimum lengths will also involve a greater peak force (or contribution to torque) by SM and BFlh than ST. Importantly, during activities such as sprint running, the muscles work high on the ascending limb and the plateau of their force-length relations, although they may work high on the descending limb in some individuals who extend the knee substantially whilst the hip is in flexion in the late swing phase (late recovery); thus the muscles should predominantly work at near-optimum lengths.

## Supplementary Information

**Additional file 1** Title of data: Model characteristics. Description of data: Summary of five models’ characteristics. Several models have used the generic model developed in openSIM [49] and, hence, it is presented first. Some models have used data and algorithms from other studies [236–239].

**Additional file 2** Title of data: Biceps femoris long head and semitendinosus knee forces and torques. Description of data: Figure displaying mean (SD) active knee flexion forces (upper graph) and torque (lower graph) of biceps femoris long head (BFlh) and semitendinosus (ST) (lower graph) at 15 different hip (H) and knee (K) flexion angles as predicted using forward simulation modeling. Joint positions are arranged from left to right, from shorter to longer muscle lengths. Values were obtained from the Lower limb model 2010 [50], the full-body running model [53], the refined musculoskeletal model [55], and the Gait2354_simbody model [49,54] and the full body model [52] using OpenSim version 4.2 software [58]. Using each model, the hamstrings muscles were fixed at five hip flexion angles (0° = neutral, −20°, 45°, 90° and 120°) and both active and passive forces and joint torques were computed at each 10° of knee joint motion from 0° (full extension) to 100° of flexion. Error bars indicate standard deviation.

**Additional file 3** Title of data: Biceps femoris long head and semitendinosus hip forces and torques. Description of data: Figure displaying mean (SD) active knee flexion forces (upper graph) and torque (lower graph) of biceps femoris long head (BFlh) and semitendinosus (ST) (lower graph) at 15 different hip (H) and knee (K) flexion angles as predicted using forward simulation modeling. Joint positions are arranged from left to right, from shorter to longer muscle lengths. Values were obtained from the Lower limb model 2010 [50], the full-body running model [53], the refined musculoskeletal model [51], and the Gait2354_simbody model [49,54] and the full body model [52] using OpenSim version 4.2 software [58]. Using each model, the hamstrings muscles were fixed at five hip flexion angles (0° = neutral, −20°, 45°, 90° and 120°) and both active and passive forces and joint torques were computed at each 10° of knee joint motion from 0° (full extension) to 100° of flexion. Error bars indicate standard deviation.

**Additional file 4** Title of data: Predicted hamstring moment-arm vs joint angle curves. Description of data: Figure displaying mean (SD) knee flexion and hip extension moment arm values of biceps femoris long head (BFlh) and semimembranosus (SM) (upper graph) and semitendinosus (ST) (lower graph) at 15 different hip (H) and knee (K) flexion angles as predicted using forward simulation modeling. Values were obtained from the full-body running model [53], the Lower limb model 2010 [50], the refined musculoskeletal model [51], and the Gait2354_simbody model [49,54] and the full body model [52] using OpenSim version 4.2 software [58]. Using each model, the hamstrings muscles were fixed at five hip flexion angles (0° = neutral, −20°, 45°, 90° and 120°) and data were obtained at each 10° of knee joint motion from 0° (full extension) to 100° of flexion. Error bars indicate standard deviation.

**Additional file 5** Title of data: Predicted peak hamstring moment-arm values. Description of data: Table displaying hip extension and knee flexion moment arm (MA) values (mm) of the hamstrings and angular position at which the maximum moment arm was observed, predicted using six models (see text for more details). Hip = hip extension angle (negative angle denotes hip extension, knee = knee flexion angle.

## Data Availability

All data generated or analyzed during this study are included in this published article.

## Abbreviations

BFlh: Biceps femoris long head
BFsh: Biceps femoris short head
SM: Semimembranosus
ST: Semitendinosus
EMG: Electromyography
PCSA: Physiological cross-sectional area
MRI: Magnetic resonance imaging
