# Supplementary material for: Hamstrings force-length relationships and their implications for angle-specific joint torques: a narrative review
Source: BMC Sports Sci Med Rehabil. 2022 Sep 5;14:166. doi: 10.1186/s13102-022-00555-6 (PMC9446565; doi:10.1186/s13102-022-00555-6)
Supplement: Supplementary file 1 — Additional file 1 Title of data: Model characteristics. Description of data: Summary of five models’ characteristics. Several models have used the generic model developed in openSIM [49] and, hence, it is presented first. Some models have used data and algorithms from other studies [236–239]. [file 13102_2022_555_MOESM1_ESM.docx]

**Appendix File 1. Table**: Summary of five models’ characteristics. Several models have used the generic model developed in openSIM [51] and, hence, it is presented first

| Study | Activity | General characteristics | Model creation parameters | Muscle parameters taken from | Force calculations | Notes |
| --- | --- | --- | --- | --- | --- | --- |
| Generic Model [51] |  | Lower body, 4 segments, 43 actuators | - Bone geometry data (determined using a three-dimensional digitizer) - Separate model for each joint - Muscle paths represented as a series of line segments - Muscle moment arms and lengths: computed using geometric equations from joint and muscle position data | 5 cadavers [45, 46, 237] | - Hill-based type model to model muscle-tendon unit properties accounting for muscle and tendon properties and scaled by peak isometric force, optimal fiber length, pennation angle and tendon slack length - Passive forces generated by muscles when they are not active and are stretched beyond their optimal length - Tendon slack length was estimated based on the relationship between fiber length, passive and active moment and joint position | |
| Lower limb model 2010 [49] | Walking | Lower body, 9 segments, 35 muscles, 44 actuators | Update on the generic model [51] | 21 cadavers [44] supplemented by other data [46, 51, 53] | - Same as generic - Maximum isometric force = PCSA times specific tension of 61 N/cm^2^. | SM fibers were short and tendon length was adjusted |
| The full-body running model [57] | Running | Lower body model, 12 segments, 76 muscles, 92 actuators | - Inverse kinematics algorithm to adjust experimental and virtual model markers - Residual reduction algorithm to compute joint moments | Based on the generic model (see above) | - Induced acceleration analysis to calculate muscle contribution to center of mass acceleration - CMC algorithm computed muscle excitations [238] |  |
| The **Gait2354_simbody model [51, 58]** | Walking | 12 segments, 92 actuators |  | Based on the generic model (see above) | - Data was reduced by making small adjustments to the model’s motion - CMC algorithm computed muscle excitations [238] | - lack of some intrinsic properties of real muscles - did not separate out the effects of force–length–velocity properties of fibres and tendon elasticity on stabilization. - Model presents some instability problems |
| London lower body model [239] | Walking,  Stair climbing | Lower body model,  6 segments, 57 muscles, 163 actuators, |  | One cadaveric specimen [52] | - Muscle sharing problem: constraints of mechanical equilibrium at the joints and physiological limits of muscle tensions [240] - Maximum isometric force = PCSA times specific tension of 37 N/cm^2^. - Inverse dynamics for determining the intersegmental moments | Contraction dynamics or force– length–velocity relationships were not implemented for the muscle actuators |
| Full body model [55] | Walking  running | Full body model, 37 DoF, 80 muscles, 97 actuators | Rigid model data from Arnold et al.  [49] | 21 cadavers [44]  and magnetic resonance images of 24 young participants | - Hill-type muscle model [241] - Optimal force from MRI muscle volumes - Maximum isometric force = PCSA times specific tension of 60 N/cm^2^ - CMC algorithm computed muscle excitations | - overestimates active and passive muscle fiber forces in some conditions - generates larger maximum isometric joint moments than real measurements |
| The refined musculoskeletal model [54] | Walking,  Running, pedaling | Full body, 80 muscles, 97 muscles | Refinement of the model by Rajagopal et al. [55] by increasing the model’s range of knee flexion, updating the paths of the knee muscles, and modifying the force-generating properties of eleven muscles. | Re-adjusted fiber lengths and tendon slack lengths of the model by Rajagopal et al. [55] | Maximum isometric force = PCSA times specific tension of 60 N/cm^2^. | - overestimates passive moments   at hip flexion > 50°   - SM and ST moment arms are too small at extreme ranges of knee flexion |

Computed muscle control (CMC) algorithm: computes muscle excitations using forward simulation which solves the muscle redundancy problem by computing muscle excitations and excitations of the ideal torque actuators that minimize an objective function [238]; PCSA = Physiological cross-sectional area; SM = Semimembranosus; ST = Semitendinosus
