# Supplementary material for: Hamstrings force-length relationships and their implications for angle-specific joint torques: a narrative review
Source: BMC Sports Sci Med Rehabil. 2022 Sep 5;14:166. doi: 10.1186/s13102-022-00555-6 (PMC9446565; doi:10.1186/s13102-022-00555-6)
Supplement: Supplementary file 2 — Additional file 2 Title of data: Biceps femoris long head and semitendinosus knee forces and torques. Description of data: Figure displaying mean (SD) active knee flexion forces (upper graph) and torque (lower graph) of biceps femoris long head (BFlh) and semitendinosus (ST) (lower graph) at 15 different hip (H) and knee (K) flexion angles as predicted using forward simulation modeling. Joint positions are arranged from left to right, from shorter to longer muscle lengths. Values were obtained from the Lower limb model 2010 [50], the full-body running model [53], the refined musculoskeletal model [55], and the Gait2354_simbody model [49,54] and the full body model [52] using OpenSim version 4.2 software [58]. Using each model, the hamstrings muscles were fixed at five hip flexion angles (0° = neutral, −20°, 45°, 90° and 120°) and both active and passive forces and joint torques were computed at each 10° of knee joint motion from 0° (full extension) to 100° of flexion. Error bars indicate standard deviation. [file 13102_2022_555_MOESM2_ESM.docx]

**
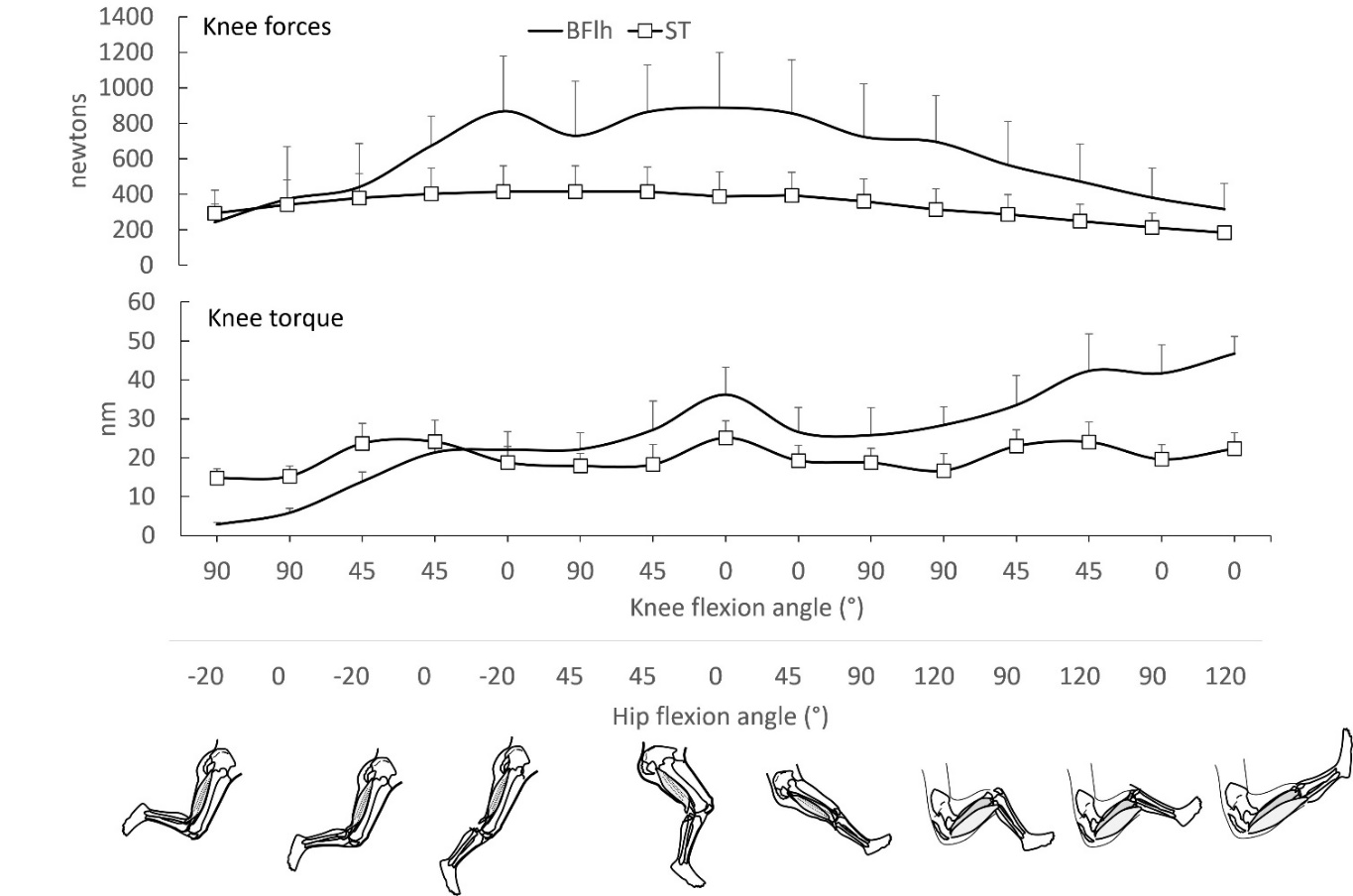
**

**Additional File 2. Figure:** Mean (SD) active knee flexion forces (upper graph) and torque (lower graph) of biceps femoris long head (BFlh) and semitendinosus (ST) (lower graph) at 15 different hip (H) and knee (K) flexion angles as predicted using forward simulation modeling. Joint positions are arranged from left to right, from shorter to longer muscle lengths. Values were obtained from the Lower limb model 2010 [49], the full-body running model [57], the refined musculoskeletal model [54], and the G**ait2354_simbody model [51, 58] and the full body model** [55] **using** OpenSim version 4.2 software [59]. Using each model, the hamstrings muscles were fixed at five hip flexion angles (0° = neutral, -20°, 45°, 90° and 120°) and both active and passive forces and joint torques were computed at each 10° of knee joint motion from 0° (full extension) to 100° of flexion. Error bars indicate standard deviation.
