# Supplementary material for: Hamstrings force-length relationships and their implications for angle-specific joint torques: a narrative review
Source: BMC Sports Sci Med Rehabil. 2022 Sep 5;14:166. doi: 10.1186/s13102-022-00555-6 (PMC9446565; doi:10.1186/s13102-022-00555-6)
Supplement: Supplementary file 4 — Additional file 4 Title of data: Predicted hamstring moment-arm vs joint angle curves. Description of data: Figure displaying mean (SD) knee flexion and hip extension moment arm values of biceps femoris long head (BFlh) and semimembranosus (SM) (upper graph) and semitendinosus (ST) (lower graph) at 15 different hip (H) and knee (K) flexion angles as predicted using forward simulation modeling. Values were obtained from the full-body running model [53], the Lower limb model 2010 [50], the refined musculoskeletal model [51], and the Gait2354_simbody model [49,54] and the full body model [52] using OpenSim version 4.2 software [58]. Using each model, the hamstrings muscles were fixed at five hip flexion angles (0° = neutral, −20°, 45°, 90° and 120°) and data were obtained at each 10° of knee joint motion from 0° (full extension) to 100° of flexion. Error bars indicate standard deviation. [file 13102_2022_555_MOESM4_ESM.docx]

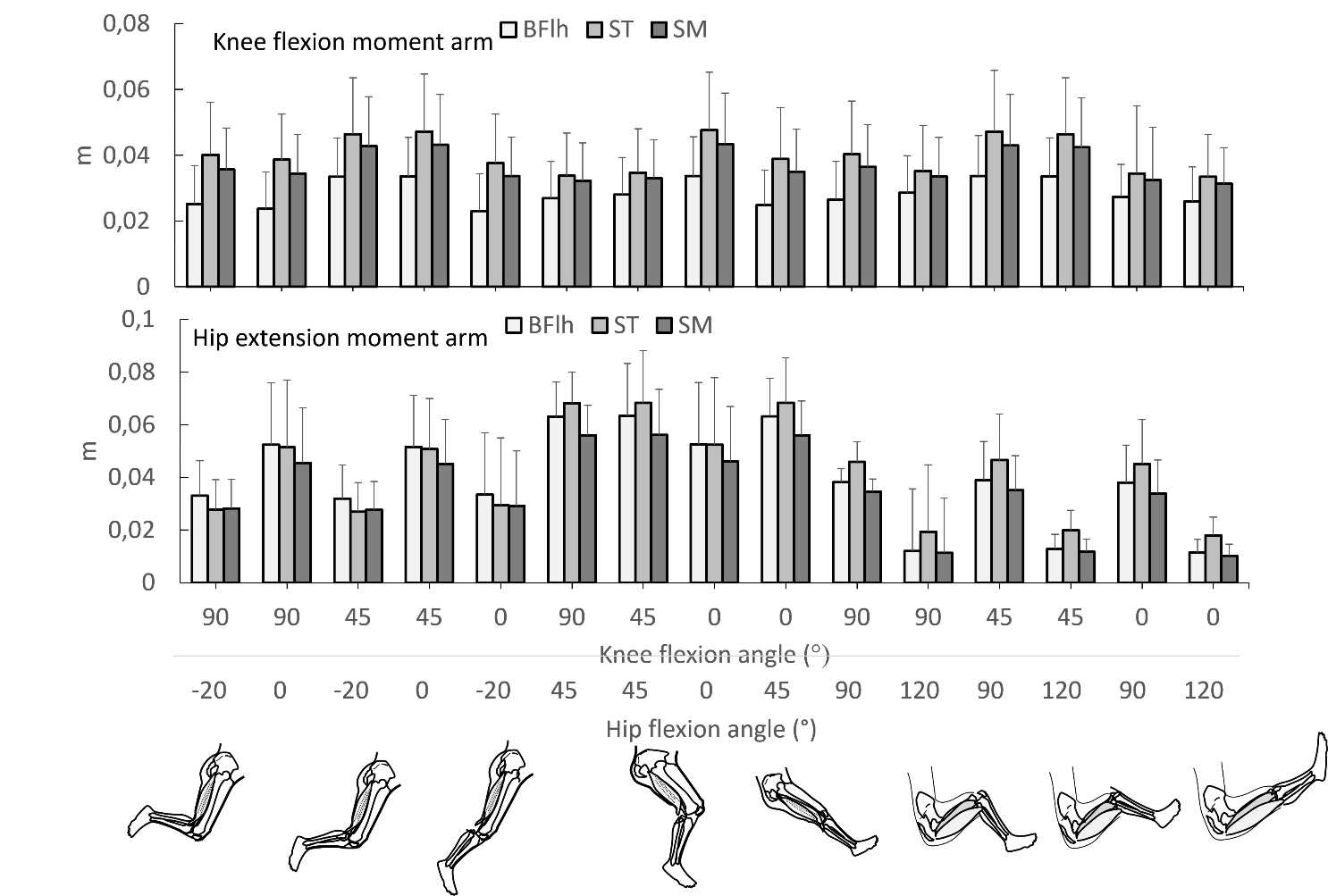


**Additional file 4 Figure:** Mean (SD) knee flexion and hip extension moment arm values of biceps femoris long head (BFlh) and semimembranosus (SM) (upper graph) and semitendinosus (ST) (lower graph) at 15 different hip (H) and knee (K) flexion angles as predicted using forward simulation modeling. Values were obtained from the full-body running model [57], the London lower limb model [239], the refined musculoskeletal model [54], and the **Gait2354_simbody model [51, 58] and the full body model [55] using** OpenSim version 4.2 software [59]. Using each model, the hamstrings muscles were fixed at five hip flexion angles (0° = neutral, -20°, 45°, 90° and 120°) and data were obtained at each 10° of knee joint motion from 0° (full extension) to 100° of flexion. Error bars indicate standard deviation.
