# Supplementary material for: Hamstrings force-length relationships and their implications for angle-specific joint torques: a narrative review
Source: BMC Sports Sci Med Rehabil. 2022 Sep 5;14:166. doi: 10.1186/s13102-022-00555-6 (PMC9446565; doi:10.1186/s13102-022-00555-6)
Supplement: Supplementary file 5 — Additional file 5 Title of data: Predicted peak hamstring moment-arm values. Description of data: Table displaying hip extension and knee flexion moment arm (MA) values (mm) of the hamstrings and angular position at which the maximum moment arm was observed, predicted using six models (see text for more details). Hip = hip extension angle (negative angle denotes hip extension, knee = knee flexion angle. [file 13102_2022_555_MOESM5_ESM.docx]

**Additional File 5.** Hip extension and knee flexion moment arm (MA) values (mm) of the hamstrings and angular position at which the maximum moment arm was observed, predicted using six models (see text for more details). Hip = hip extension angle (negative angle denotes hip extension, knee = knee flexion angle.

|  | Biceps femoris long head | | Semimembranosus | | Semitendinosus | |
| --- | --- | --- | --- | --- | --- | --- |
|  | Hip extension | | | | | |
|  | Hip flexion (°) | MA (mm) | Hip flexion (°) | MA (mm) | Hip flexion (°) | MA (mm) |
| Lower limb model 2010 [49] | 30 | 62.16 | 30 | 55.5 | 30 | 66.6 |
| The full-body running model [57], The **Gait2354_simbody model [51, 58]** | 30 | 65.81 | 30 | 57.7 | 30 | 70.7 |
| Full body model [55] | 40 | 62.2 | 40 | 55.3 | 40 | 66.7 |
| The refined musculoskeletal model [54] | 40 | 62.0 | 40 | 55.5 | 40 | 66.7 |
|  | Knee flexion | | | | | |
|  | Knee flexion (°) | MA (mm) | Knee flexion (°) | MA (mm) | Knee flexion (°) | MA (mm) |
| Lower limb model 2010 [49] | 50 | 37.15 | 50 | 46.45 | 60 | 55.34 |
| The full-body running model [57], The **Gait2354_simbody model [51, 58]** | 30 | 34.93 | 50 | 40.49 | 50 | 48.04 |
| Full body model [55] | 50 | 37.27 | 50 | 46.23 | 60 | 55.33 |
| The refined musculoskeletal model [54] | 70 | 31.31 | 60 | 37.77 | 50 | 48.53 |
